# Effect of Nanoceria Suspension Addition on the Physicochemical and Mechanical Properties of Hybrid Organic–Inorganic Hydroxyapatite Composite Scaffolds

**DOI:** 10.3390/nano14131102

**Published:** 2024-06-27

**Authors:** Paraskevi Gkomoza, Ioanna Kitsou, Savvas Koltsakidis, Dimitrios Tzetzis, Andreas Karydis-Messinis, Nikolaos Evangelos Zafeiropoulos, Foteini Gerodimou, Eleni Kollia, Vasilis Valdramidis, Athena Tsetsekou

**Affiliations:** 1 Laboratory of Metallurgy, School of Mining & Metallurgical Engineering, National Technical University of Athens, 9 Heroon, Polytechniou Ave., 15772 Zografos, Athens, Greece; pgomoza@metal.ntua.gr (P.G.); kitioanna@metal.ntua.gr (I.K.); 2 Digital Manufacturing and Materials Characterization Laboratory, School of Science and Technology, International Hellenic University, 14th km Thessaloniki—N. Moudania, 57001 Thermi, Thessaloniki, Greece; skoltsakidis@ihu.edu.gr (S.K.); d.tzetzis@ihu.edu.gr (D.T.); 3 Department of Material Science and Engineering, University of Ioannina, 45110 Ioannina, Greece; a.karyd@uoi.gr (A.K.-M.); nzafirop@uoi.gr (N.E.Z.); 4 Laboratory of Food Chemistry, Department of Chemistry, National and Kapodistrian University of Athens, 15771 Zografos, Athens, Greece; foteiniger@chem.uoa.gr (F.G.); elenikollia@chem.uoa.gr (E.K.); valdram@uoa.gr (V.V.)

**Keywords:** hydroxyapatite scaffolds, CeO_2_, DMA, mechanical properties, antifungal properties

## Abstract

In the current study, the synthesis of hydroxyapatite-ceria (HAP-CeO_2_) scaffolds is attempted through a bioinspired chemical approach. The utilized colloidal CeO_2_ suspension presents antifungal activity against the *Aspergillus flavus* and *Aspergillus fumigatus* species at concentrations higher than 86.1 ppm. Three different series of the composite HAP-CeO_2_ suspensions are produced, which are differentiated based on the precursor suspension to which the CeO_2_ suspension is added and by whether this addition takes place before or after the formation of the hydroxyapatite phase. Each of the series consists of three suspensions, in which the pure ceria weight reaches 4, 5, and 10% (by mass) of the produced hydroxyapatite, respectively. The characterization showed that the 2S series’s specimens present the greater alteration towards their viscoelastic properties. Furthermore, the 2S series’s sample with 4% CeO_2_ presents the best mechanical response. This is due to the growth of needle-like HAP crystals during lyophilization, which—when oriented perpendicular to the direction of stress application—enhance the resistance of the sample to deformation. The 2S series’s scaffolds had an average pore size equal to 100 μm and minimum open porosity 89.5% while simultaneously presented the lowest dissolution rate in phosphate buffered saline.

## 1. Introduction

As is known, most composite materials show combined plastic deformation and fracture during failure. Crack propagation in ceramic materials tends to advance swiftly after crack initiation. Therefore, crack initiation needs to be prevented through mitigation of concentrated stresses in the entire volume of the material and also via reinforcement of the main material phase [1]. Secondary phase structures in the form of either particles or fibers, when in dispersion throughout the parent phase, can act as multiple points of local stress concentration. Therefore, concentration of stresses at one point of the material is avoided and dislocation slip is discouraged. Even in the case of crack initiation, cracks are intercepted due to successive deflections in the failure propagation path at the crystal boundaries in a polycrystalline material.

The secondary reinforcing phase in a ceramic composite most commonly refers to oxides or carbides, which may be present in a lower (3–5%) or higher (12–15%) percentage within the matrix phase [2]. For example, yttria-stabilized zirconia and tetragonal zirconia powders were used for the fabrication of composite hydroxyapatite-zirconia nanostructures, in which the percentage of the zirconia phase ranged between 1.5 and 15% [3]. HAP synthesis was carried out inside a colloidal suspension of the respective zirconia powder and the precipitated material was calcined at 550 °C for 2 h. The produced nanocomposites mechanically outperformed both the pure nanohydroxyapatite powder and also the conventional hydroxyapatite-based composites. In a more recent study [4], HAP composite powder doped with metallic cerium atoms was synthesized through a wet precipitation process at 40 °C. After the precipitated paste was dried at 100 °C for 24 h, a quantity of the dried powder was compressed into a pellet and fired for 2 h at 1250 °C. After mechanical characterization of the composite material, it was concluded that for doping with cerium content up to 1.5% wt., the produced composite reached its highest density and presented a microhardness value of 42.3 ± 6.2 GPa. In both the above cases, the manufactured composite materials were considered suitable for use in biomedical applications as bone graft materials. However, in the present work, although calcination of the ceramic pastes could potentially enhance their mechanical properties, it is avoided due to the presence of integrated biomolecules that are intended to improve their bioactivity.

Interconnected porous microstructure of the fabricated scaffolds is one of the most desirable microstructural features if osteogenesis is desired. The internal porosity of the scaffolds can be divided into three categories—namely macroporosity for cases in which the total number of pores exceeds 100 μm, microporosity when the size distribution of the pores varies between 0.1–10 μm, and nanoporosity for contained pores smaller than 0.1 µm [5]. For the successful migration and movement of cells through the scaffold, the required average size of the pores in its internal porous network starts from 100 μm up to about 350 μm (or greater) [6]. However, recent studies examine the impact that varying or combining pore size distributions have on the osteogenic effects of said pores. Particularly, gradient porosity can contribute to hindering the diffusion of cell nutrients from the scaffold and therefore their retention inside the scaffold for a longer time period [7]. Numerous data from the literature support the existence of a direct correlation between the porosity content and the mechanical properties of the scaffolds, according to recent review [8]. Specifically, the mechanical properties undergo exponential degradation as the porosity content increases. Furthermore, the mechanical response of the scaffolds can be affected by the uniformity of the pores, their shape and orientation, apart from their size. Calcination of the ceramic pastes could, potentially, lead to a densification of the material and result in less porous structures, through the sintering step. However, as mentioned above, the existence of porosity is of vital importance for osteoblast adhesion and proliferation, and consequently long-term cell viability. For this reason, mechanical reinforcement of the scaffolds is attempted through the dispersion of a secondary phase of ceria (CeO_2_) nanoparticles in the initial bioceramic–biopolymer composite phase. CeO_2_ is crystallized at ambient temperature, and thus, its incorporation into the composite HAP–biopolymer matrix in the form of a suspension is enabled. In this way, the formation of large aggregations and the consequent disruption of the internal microstructure of the scaffolds as well as degradation of both physicochemical and mechanical properties are avoided.

Chitosan-based scaffolds can speed up the creation of osseous tissue by acting as transient framework for osteogenic cell activity, since chitosan is characterized by excellent osteoconductivity, encourages osteogenic differentiation and mineralization, and inhibits inflammatory reactions. Because of these characteristics, chitosan-based scaffolds are desirable option for use in orthopedic and regenerative bone treatment applications. When calcium phosphates, such as hydroxyapatite, are intertwined in the biopolymer phase, the mechanical and biological characteristics of the hybrid scaffold are further enhanced [9]. Moreover, in this particular project, a biomimetic synthesis route is followed, as the synthesis is complete in an L-arginine environment. In this way, the produced calcium phosphate phase constitutes hydroxyapatite with close proximity to biological apatite, regarding its physicochemical properties. In addition, it is widely known that chitosan presents excellent antifungal activity, which can be mediated by numerous physicochemical parameters such, as its physical state (e.g., solution/particle form), molecular weight, degree of deacetylation (DD), and pH [10]. However, although higher antifungal activity is expected from highly deacetylated chitosan, no antifungal activity was detected against either species of *Aspergillus fumigatus* or *Aspergillus parasiticus* when chitosan with a DD equal to 98% was tested, even at high concentrations of 2000 ppm [11]. Additionally, the antifungal activity of chitosan of different molecular weights but with a high DD of 95% was investigated [12]. It was concluded that for intermediate molecular weights in the range of 140–200 kDa, antifungal activity was diminished, while for molecular weights beyond 800 kDa, the development of fungus was even enhanced. In another study, both chitosan solution and nanoparticles derived from chitosan with a molecular weight equal to 310 kDa and a DD in the range of 75 to 85% achieved inhibition of *Aspergillus niger* at concentrations higher than 1.7 mg/mL (≈1700 ppm) [13]. This is due to the inherent resistance of *Aspergillus niger* to chitosan, since chitin is already present in the cell wall of the fungus at a 10% rate [13]. As a result, antifungal protection should be enhanced as far as *Aspergillus* species are concerned.

For this reason, the potential antifungal activity of CeO_2_ nanoparticles was investigated. Through extensive literature survey, it was found that there is a significant lack of reports on the antifungal properties exhibited by ceria, particularly when CeO_2_ nanoparticles are incorporated into complex three-dimensional calcium phosphate structures. In previous research [14], suspensions of hydroxyapatite doped with cerium were synthesized via a modified chemical co-precipitation synthesis method and respective coatings were manufactured with the use of the produced powders through radio frequency magnetron sputtering. After in vitro antimicrobial assessment was performed, it was found out that both the original suspensions and the coatings hindered microbial growth after the first day of incubation, while the original suspensions presented biocidal action beyond three days of incubation against *Escherichia coli* and *Candida albicans* strains. The integrated cerium ions in the hydroxyapatite crystal structure were considered responsible for the antimicrobial properties of the composite materials, due to their ability to affect production and regulation of reactive oxygen species and therefore, lead to microbial cell death in the long term. In a similar and more recent study [15], a similar cerium-doped hydroxyapatite suspension was synthesized via the sol–gel synthesis method and then spin-coated onto a silicon substrate. Again, both the original suspension and the coating presented biocidal action beyond three days of incubation against *Candida albicans* strain. Particularly, the composite substrate totally suspended fungal biofilm formation. Apart from the established antimicrobial properties of CeO_2_ nanoparticles, the possibility that they boost bone tissue regeneration also constitutes an intriguing issue. In a recent study [16], composite chitosan-based iron-doped dicalcium phosphate dihydrate (Brushite) scaffolds were manufactured in which a content of cerium oxide nanoparticles between 10 and 30 wt.% had been incorporated. The ingredients were stirred under heating until the mixture was completely homogenized. Then, the composite material was frozen at −80 °C and subsequently underwent lyophilization at −100 °C. Examination of the scaffolds revealed that the number of pores increased dramatically while the distribution of pore sizes shrank, as CeO_2_ nanoparticle concentrations rose. The increase in the surface roughness of the samples was also proportional to the CeO_2_ content integrated in the samples. Indirect cytotoxicity studies suggested that both the size and number of the pores in the scaffolds are adequate for cell growth, which is further reinforced by the morphology of the scaffolds. Therefore, the use of CeO_2_ nanoparticles is speculated to attribute antifungal properties to the hybrid scaffolds and therefore, making them a promising biomaterial for use in dental procedures or in cases when preventive administration of antifungal drugs is recommended (e.g., immunosuppressed people).

In the current study, the synthesis of optimized composite hydroxyapatite-ceria (HAP-CeO_2_) scaffolds is attempted, with the addition of a colloidal CeO_2_ suspension with confirmed antifungal activity. The ultimate goal is the construction of composite highly porous HAP scaffolds, in which dispersion of the CeO_2_ phase within the hybrid organic-inorganic matrix phase has been achieved and which present enhanced physicochemical and mechanical properties. Based on a preliminary research effort performed in the laboratory, the manufacture of HAP-CeO_2_ scaffolds in which the weight of the incorporated CeO_2_ nanoparticles ranged from 1 to 5% (by mass) of the produced hydroxyapatite was achieved. Examination of the viscoelastic properties of these scaffolds revealed that the dynamic mechanical response of the scaffolds with CeO_2_ content equal to 4 wt.% and 5 wt.% was enhanced compared to the response of the scaffolds with CeO_2_ content equal to 1, 2, and 3 wt.%. Thus, the study was focused on the development of HAP-CeO_2_ scaffolds with CeO_2_ content equal to 4 and 5 wt.%. Additionally, scaffolds with CeO_2_ content equal to 10 wt.% were studied, so comparison between scaffolds with low and high percentages of reinforcing phase is possible.

## 2. Materials and Methods

### 2.1. Materials

For the hydroxyapatite scaffolds, calcium hydroxide (Ca(OH)_2_) was purchased from Lach-Ner, Ltd. (Lach-Ner, Ltd., Neratovice, Czech Republic). and orthophosphoric acid (H_3_PO_4_, 85+%) was acquired from Fisher Scientific. The added biopolymers chitosan of high molecular weight (C_12_H_24_N_2_O_9_, Mr ≈ 310,000–375,000 Da, DD ≥ 75%) and L-arginine (C_6_H_14_N_4_O_2_, reagent grade, ≥98%) were both acquired from Sigma-Aldrich (Sigma-Aldrich Biochemie GmbH, Hamburg, Germany).

For the ceria suspension, cerium (III) nitrate hexahydrate (Ce(NO_3_)_3_·6H_2_O) salt was purchased from Sigma-Aldrich, polyethylene glycol (PEG, HO-(-CH_2_CH_2_O)n-H, Mr ≈ 200, n ≈ 4) was provided by SERVA FEINBIOCHEMICA GmbH & Co. KG (Heidelberg, Germany), and high-purity PATris(tris(hydroxymethyl)aminomethane (Tris) buffering solution was acquired from MP Biomedicals LLC (MP Biomedicals Germany GmbH, Eschwege, Germany). Distilled water was used as a solvent throughout the whole process.

### 2.2. Synthesis of the HAP-CeO_2_ Suspensions

#### 2.2.1. Synthesis of Ceria Colloidal Suspension

The synthesis method of the ceria suspension has already been presented in detail in previously published research [17]. Briefly, an aqueous cerium nitrate salt solution was prepared. The initial quantity of cerium nitrate salt was weighed according to the analytically calculated target mass of the produced ceria in the final ceria suspension. In total, three different ceria suspensions were synthesized, in which the pure ceria weights were 4, 5, and 10% (by mass) of the produced hydroxyapatite in the control suspension, respectively. Then, 1 mL of PEG was added dropwise to the starting cerium nitrate solution under continuous magnetic stirring. Upon dissolution of PEG, Tris base buffer 20% *w*/*v* aqueous solution was also added dropwise under continuous magnetic stirring until the pH of the final suspension reached the value of 10.2. After pH stabilization, the final suspension was left to be magnetically stirred for the next 24 h. The synthesis process was performed at ambient temperature.

#### 2.2.2. Synthesis of the Hydroxyapatite Suspension

For the synthesis of the hydroxyapatite suspensions a biomimetic approach was followed based on previously published laboratory research [18]. This process is carried out through successive crystallization, precipitation and dissolution of intermediate calcium phosphate phases of high solubility, which constitute precursor HAP phases. The formation and solubility of each phase is dependent on the pH value of the solution and is affected by the saturation of the system. Upon completion of the process, an HAP phase is formed, which constitutes the most stable calcium phosphate phase and has low solubility. In brief, a suspension of calcium hydroxide [0.25 M] (pH ≈ 12.0–12.3), a dilute aqueous solution of orthophosphoric acid [0.15 M], and a solution of L-arginine [0.5 M] (pH ≈ 11.3) were prepared. The Ca(OH)_2_ suspension was mechanically stirred at 3200 rpm for 15 min, and it was then vigorously stirred with a magnetic stirrer until the end of the process. An amount of chitosan equal to a quarter of the total L-arginine amount was mechanically dissolved in the H_3_PO_4_ solution at 8200 rpm for 30 min inside an ice bath. The chitosan solution (pH ≈ 1.5) was then magnetically stirred at room temperature until the end of the process. Both the Ca(OH)_2_ and the chitosan solution were simultaneously added dropwise into the L-arginine solution, which was magnetically stirred at 40 °C inside an aqueous bath. The pH value of the L-arginine solution gradually decreased during the dropwise addition of the reactants, until it was stabilized at about 10.25. After HAP formation, the pH value in the final suspension can affect the adsorption mechanism of other compounds on the surface of the HAP crystals. Different surfaces of the HAP crystal are positively or negatively charged according to the pH value in the final suspension. Thus, when compounds with one or more functional groups in their molecule are present, the conjugation due to electrostatic interaction is performed among different functional ends and on different surface sites of the HAP crystal. Therefore, controlling the pH during HAP synthesis enables oriented HAP crystal growth.

For the synthesis of the composite HAP-CeO_2_ suspensions, three different suspension series were produced, namely 1S, 2S, and 3S. In each of these series, the entire volumes of the priorly prepared CeO_2_ suspensions were used, with the volume ratio of HAP to CeO_2_ suspensions being 1:1. The differentiation among the series of suspensions is based on the precursor suspension to which the CeO_2_ suspension was added and by extension whether this addition takes place before or after the formation of the hydroxyapatite phase. In detail, 1S refers to the composite HAP-CeO_2_ suspensions, in which the CeO_2_ suspension was added after the synthesis of the hydroxyapatite in the HAP suspension; 2S refers to the composite HAP-CeO_2_ suspensions, in which respective volume (mL) of CeO_2_ suspension was used as a solvent for the arginine solution instead of H_2_O. Subsequently, equal volumes of Ca(OH)_2_-(H_3_PO_4_-Chit) precursor suspensions and CeO_2_ suspension were alternately added to the arginine solution. In this way, HAP synthesis was achieved within the CeO_2_ suspension. Finally, 3S refers to the composite HAP-CeO_2_ suspensions, in which respective volume (mL) of CeO_2_ suspension was used as a solvent for both the arginine solution and the Ca(OH)_2_ suspension, instead of H_2_O. The remaining quantity (mL) of CeO_2_ suspension was added after the entire volumes of the respective precursors Ca(OH)_2_ and (H_3_PO_4_-Chit) had already been used. The pH values of the HAP-CeO_2_ suspensions did not greatly vary from the pH value of the HAP suspension since the added CeO_2_ suspensions have a pH value of about 10.2. The final suspensions were aged at 40 °C for 3 h. The code names of the examined compositions are presented in the following table (Table 1).

### 2.3. Manufacture of the Composite HAP-CeO_2_ Scaffolds

The final composite suspensions were centrifuged in a benchtop centrifuge (type Z326, HERMLE Labortechnik GmbH, Wehingen, Germany) at 11,000 rpm for 15 min. After the supernatant solvent was disposed of, washing of the precipitated pastes followed at 11,000 rpm for another 15 min with water as a solvent. In this way, excess biopolymer along with remaining salt and starting compound traces were removed. The collected paste material was then spread inside specifically designed cylindrical perforated silicone molds. The cast of the molds had been 3D-printed with a Photon Mono SE (Anycubic) 3D-printer, provided for use by the company Nanotypos (Nanotypos Advanced Nanomanufacturing, Thessaloniki, Greece). Next, the pastes underwent freezing at ≈−50 to −55 °C for 3 h and then drying under vacuum for 24 h so that removal of excess moisture from the samples was achieved. The used apparatus was a benchtop freeze-dryer (type LyoQuest, Telstar, Barcelona, Spain). The produced samples had a diameter of 15 mm and a thickness of 10 mm.

### 2.4. Characterization Techniques

In order to properly characterize the manufactured composite hydroxyapatite scaffolds, the following methods were utilized: scanning electron microscopy (SEM), transmission electron microscopy (TEM), X-ray diffraction (XRD), and Fourier-transform infrared spectroscopy (FTIR). The mechanical properties of the scaffolds were evaluated via dynamic mechanical analysis (DMA), uniaxial compression testing (UCT), and cyclic loading. Furthermore, scaffold porosity measurements were performed. Additionally, the antifungal activity of the CeO_2_ suspension was evaluated.

#### 2.4.1. Microstructural Characterization

A scaffold of each composition was transversely cut. The smaller pieces of the original scaffolds were coated with a thin layer of gold so that they were electrically conductive. An electric current of 40 mA was applied for 1 min in a sputter coater (Automatic Sputter Coater, Agar Scientific, Essex, UK) under a pressure of around 0.05 mbar. After the gold sputtering was complete, the microstructure of the specimens was observed with a scanning electron microscope (type JEOL SEM 6380LV, JEOL Ltd., JEOL Europe BV, Zaventem, Belgium) at high vacuum mode.

Parts of the dried scaffolds were ground into fine powders, which were then sieved. A small amount of each sample was dispersed in pure ethanol. The suspensions remained in an ultrasonic bath for a period of 10 min, so that the powders are sufficiently dispersed. A drop of each suspension was placed on a special copper grid and was then left to dry. The samples were examined with a high-resolution transmission electron microscope (type HR-TEM, JEOL JEM-2100, JEOL Ltd., JEOL Europe BV, Zaventem, Belgium).

The XRD spectra of the powders were acquired via a Bruker D8 Focus X-ray Diffractometer over a 2θ range of 10–80° at a scan rate of 0.02° s^−1^, using CuKα1 (λ = 1.5406 Å) monochromatic radiation generated at 40 kV and 40 mA. Identification of the formed phases was achieved through Crystallographica Search-Match software (version 3.0.1.1 with RDB support) that holds a built-in JCPDS card database.

FTIR spectra were acquired using a Jasco 4200 FT-IR spectrometer (Jasco, Tokyo, Japan), in the 4000–400 cm^−1^ range. Traces of the respective scaffold powder were mixed with a proper amount of potassium bromide and then they were pressed together in order to form a pellet. Afterwards, each pellet was placed inside the spectrometer, and the acquired spectra of each of the series were compared to a HAP reference spectrum.

#### 2.4.2. Viscoelastic Properties

The dynamic mechanical response of the dry scaffolds was recorded using a dynamic mechanical analyzer (DMA Q800, T.A. Instruments, Leuven, Belgium) in compression mode under heating. The temperature ranged from 30 °C to 120 °C with a temperature ramp of 2 °C/min and an applied frequency of 1 Hz. The viscoelastic characteristics of the samples determined were the storage modulus E′ (MPa), the loss modulus E″ (MPa) and the loss factor or otherwise damping tanδ, which were compared to the corresponding values of a HAP reference scaffold.

#### 2.4.3. Mechanical Characterization

The samples (n = 3 for each composition) were subjected to unconfined uniaxial compression testing (UCT) under ambient conditions via a TESTOMETRIC M500-50AT tensile machine (The Testometric Co., Ltd., Rochdale, UK). The descent speed of the head was selected equal to 1 mm/min. The displacement (mm) data were recorded as a function of the load (N) and of the time of measurement. From the load–displacement data, the stress (MPa)–compressive strain curves were plotted. The modulus of compression Ε_i_ of each of the tested scaffolds i (i = 1, 2, 3) (MPa) was calculated as the maximum slope of the respective stress–strain curve in a 3% strain and within a 0–20% strain [19,20]. The energy absorption per unit volume EA_i_ of the examined scaffolds was also calculated (mJ/mm^3^) through the method of discrete integrals as the area enclosed by the stress–strain curve and the strain axis [21,22] and for strain equal to 50% [23].

Finally, the scaffolds (n = 1 for each composition) were subjected to cyclic uniaxial compression up to 50% strain for 5 cycles. Once again, the displacement (mm) data were recorded as a function of the load (N) and of the time of measurement, and the stress (MPa)-compressive strain curves were plotted. The area enclosed by the loading and unloading curves for each cycle i (i = 1, 2, 3, 4, 5) is the plastic deformation energy per unit volume PD_i_ of the material and represents the plastic energy absorbed by the material for its deformation caused by the applied load. The area below the loading–unloading loop, enclosed by the unloading curve and the strain axis at the point of maximum applied stress (maximum strain) for each cycle I, is known as the elastic recovery energy per unit volume ER_i_ of the material and represents the recovered work of elastic forces during unloading. Both PD_i_ and ER_i_ were calculated (mJ/mm^3^) through the method of discrete integrals.

#### 2.4.4. Scaffold Porosity Measurements

Porosity percentage of the scaffolds (n = 2 for each composition) was calculated through the Archimedes method based on the corresponding ASTM standard for measuring porosity in refractory materials [24] and adapted so the scaffolds were protected from the intense boiling conditions. Firstly, the dry scaffold mass W_d_ (g) was measured using a digital mass balance (±0.0001 g). Then, the scaffolds were immersed into isopropanol for five minutes. Subsequently, the weights of the samples under buoyancy (suspended weight) W_sus_ (g) and their weight immediately after the surface amount of water was removed using filter paper (saturated weight) W_sat_ (g) were recorded. Based on the recorded values, the percentage of open porosity P and the density of the porous samples d were calculated as:

P (%) = [(W_sat_ − W_d_)/(W_sat_ − W_sus_)] × 100, (1)

and

D = W_d_/(W_sat_ − W_sus_) (2)

#### 2.4.5. Swelling, Absorption, and Dissolution Assessment

The water uptake capacity of the scaffolds was assessed by determining their swelling and absorption ability, respectively [25]. The scaffolds (n = 2 for each composition) were weighed and their dry scaffold mass W_d_ (g) was recorded. Then, the scaffolds were immersed into 15 mL of a buffer solution of phosphate ions (PBS, pH = 7.4) for 24 h at 37 °C. The samples were withdrawn, and their mass in g was recorded before and immediately after the surface amount of water was removed using filter paper, W_s_ (swollen weight) and W_sat_ (saturated weight), respectively. The samples were immersed again into the PBS solution and the same procedure was repeated after 48 and 72 h. The volume of the buffer was made up to 15 mL if needed. The swelling ratio SR_i_ (%) and the absorption ratio AR_i_ (%) of each scaffold after i hours (i = 24, 48, 72) were calculated as:

SR_i_ (%) = [(W_s_ − W_d_)/W_d_] × 100, (3)

and

AR_i_ (%) = [(W_sat_ − W_d_)/W_d_] × 100 (4)

Slow dissolution of the scaffolds in PBS is considered an indication of slow in vitro dissolution, since the buffer systems functioning in blood plasma also include phosphate buffers. Slow dissolution of the scaffolds is desirable in order for the bone graft to maintain its shape and integrity until complete osseointegration. The dissolution of the scaffolds was assessed by determining their mass loss after immersion and retention into the PBS solution for 7 days at 37 °C. After a week, the aforementioned scaffolds were retrieved, washed with distilled water in triplicate and were subjected to lyophilization. Their weight after the freeze-drying process W_fd_ (g) was recorded. The percentage of dissolution D of each scaffold was calculated as:

D (%) = [(W_d_ − W_fd_)/W_d_] × 100 (5)

### 2.5. Antifungal Study on CeO_2_ Suspension

The antifungal properties of CeO_2_ nanoparticles were investigated through in vitro analysis in cultures of *Aspergillus* spp., namely *Aspergillus flavus* and *Aspergillus fumigatus*, that were acquired from the Faculty of Health Sciences (University of Malta). Initially, two different inocula were prepared. In detail, the fungal species were cultured on substrates containing Malt Extract Agar (MEA, NEOGEN) nutrient medium and were incubated for 5 days at 25 °C. Then, 10 mL of sterile distilled water along with tween-80 was added to the cultures. The conidia were collected after filtering through quadruple gauze, and 4 10-fold dilutions were conducted until a level of 100 conidia/10 µL was reached which was verified with Neubauer hematocytometer. The inocula were kept at 4 °C until use.

Different concentrations of CeO_2_ nanoparticles, from 0 to 356 ppm, were added in 12-well cell plates mixed with 1 mL of nutrient material, and 10 μL of inocula was added to each well. The plate with the respective fungal culture was incubated for 6 days at 25 °C. The minimum inhibition concentration (MIC) of CeO_2_ nanoparticles was determined, and their corresponding antifungal action were evaluated macroscopically, i.e., inhibition/no inhibition.

## 3. Results and Discussion

### 3.1. SEM

**Figure 1:**
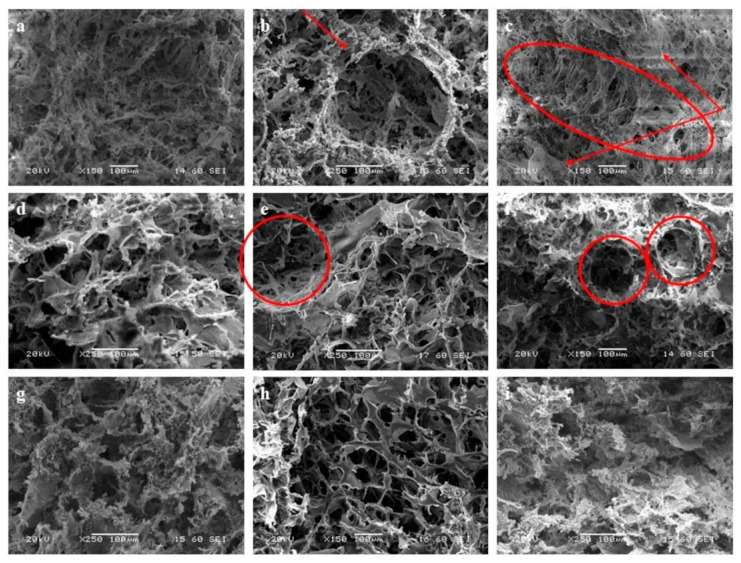
SEM images of the composite HAP–CeO_2_ scaffolds. (**a**) 1S—4% CeO_2_, (**b**) 1S—5% CeO_2_, (**c**) 1S—10% CeO_2_, (**d**) 2S—4% CeO_2_, (**e**) 2S—5% CeO_2_, (**f**) 2S—10% CeO_2_, (**g**) 3S—4% CeO_2_, (**h**) 3S—5% CeO_2_, (**i**) 3S—10% CeO_2_.

The internal microstructures of each of the examined 1S, 2S, and 3S scaffolds are presented below in the acquired SEM images (Figure 1). Two periodic zones of porosity are distinguished for the 1S—10% CeO_2_ sample, one of which is characterized by elongated ellipsoidal pores up to 100 μm in length (Figure 1c, indicated with an ellipsoidal shape of red outline) and a second one that constitutes a denser zone of 20 µm pores (Figure 1c, indicated with red arrows). Although this could be a result of the mold-filling process, the periodic character of this microstructural feature needs to be interpreted in conjunction with the findings of other characterization methods. Moreover, aggregates of calcium compounds are observed for the 1S—4% CeO_2_ (Figure 1a and Figure S1c) and 1S—5% CeO_2_ (Figure 1b and Figure S1f) scaffolds, the development of which is less intense for the 1S—10% CeO_2_ sample (Figure S1i).

The pores of the 2S series’s scaffolds (Figure 1d–f) follow a more defined pattern compared to those of the 1S series’s scaffolds (Figure 1a–c). They are nearly circular in shape and form a dense interconnected network that consists of thin chitosan walls, on which nucleated HAP nanoparticles are integrated [9,26,27]. Mineralized HAP nanoparticles are more easily distinguished in the 2S—10% CeO_2_ microstructure (Figure S2i, red arrow indication) than on the smooth-surfaced chitosan walls of the 2S—4% CeO_2_ (Figure S2c) and 2S—5% CeO_2_ (Figure S2f) scaffolds. Some much larger circular pores are discerned among the network of pores of the 2S series’s scaffolds, which reach a diameter of 120–130 µm (Figure 1e,f, red-colored circular indications). These pores are the result of salt solution crystallization at specific points, which act as porogens [27]. In our case, a large amount of Tris salt is dissolved in the final suspension, which may not be removed completely during washing of the ceramic paste and is subsequently solidified during the freezing stage. As a result, larger pores are formed after sublimation of the salt solution crystals, during the drying stage of the ceramic paste. Such pores are seldom observed in the microstructure of 1S—5% CeO_2_ scaffold (Figure 1b, indicated with a red arrow).

Regarding the microstructure of the 3S series’s scaffolds (Figure 1g–i), 3S—10% CeO_2_ scaffold (Figure 1i) is characterized by the densest microstructure, with the distributed pores ranging from 20 to 70 μm and occasionally, up to 100 μm. The porous network of 3S—4% CeO_2_ scaffold (Figure 1g) is more open with slightly larger pores ranging between 30 and 100 µm that sometimes reach a diameter of 110 µm. The 3S—5% CeO_2_ scaffold (Figure 1h) exhibits the highest degree of microstructural uniformity among the 3S series’s scaffolds since the enclosed pores are almost circular in shape. The pores fall into two size distributions, the first of which consists of large pores in the range of 70 to 100 µm and the second of which contains slightly smaller pores with an average size of 50 μm. Finally, the 3S—5% CeO_2_ scaffold is characterized by smooth-surfaced pore walls, in which nucleation of the HAP phase in the nanoscale has occurred (Figure S3f). On the contrary, mineralization and growth of a calcium phase, integrated within the porous walls of the scaffolds, is more intense for the 3S—4% CeO_2_ (Figure S3c) and 3S—10% CeO_2_ (Figure S3i) scaffolds. Nucleation of both the HAP and the calcium-deficient hydroxyapatite (CDHA) phases in the scaffolds are verified by spot EDS analyses with SEM (Figure S4).

### 3.2. TEM

**Figure 2:**
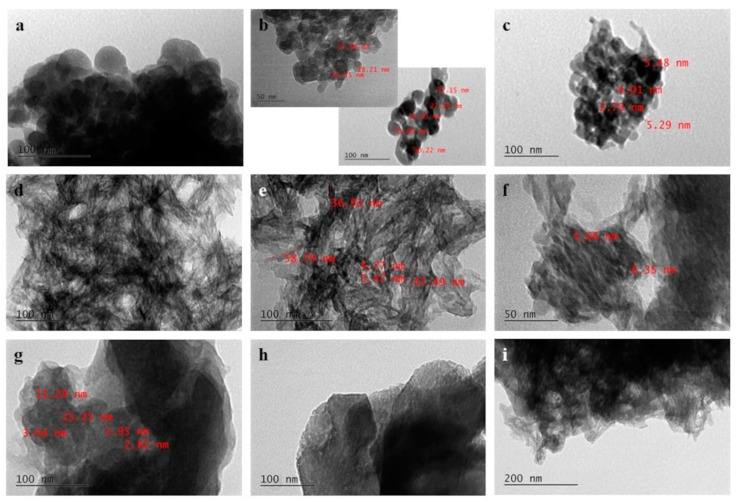
TEM images of the composite HAP–CeO_2_ scaffolds. (**a**–**c**) 1S—4% CeO_2_, (**d**–**f**) 2S—4% CeO_2_ and (**g**–**i**) 3S—4% CeO_2_.

Concerning the 1S series’s sample, it is observed that the entire particle structure consists of perfectly spherical crystallites (Figure 2a) of two size groups with respective average size of 20 nm and 33 nm, well dispersed among themselves (Figure 2b). CeO_2_ nanoparticles with an average size of 5 nm are also discerned (Figure 2c). On the contrary, the 2S series’s sample consists of needle-like HAP crystals (Figure 2d) with a length that ranges between 37 and 59 nm and a thickness that does not exceed 2–4 nm (Figure 2e). Once again, CeO_2_ nanocrystals with an average size of 5 nm (Figure 2g), are noticed to be integrated amongst the HAP crystals (Figure 2d,e). The 3S series’s sample presents a more complex nanostructure (Figure 2g), as both needle-like HAP crystals (Figure 2i) and rod-like crystals with average dimensions 12 nm long and about 5 nm thick are spotted (Figure 2h). The rod-like crystals coalesce into larger aggregates with an average size of 200 nm (Figure 2h). CeO_2_ nanocrystals are difficult to distinguish (Figure 2g), probably due to the presence of excess polymer in the sample.

As can be seen in the TEM images above, the observed HAP nanoparticles greatly vary for each of the 1S, 2S, and 3S series. This is because arginine is absorbed into specific crystalline planes of the HAP crystallites and therefore acts as a growth inhibitor, allowing the crystallites to grow towards specific directions. More specifically, L-arginine molecules are positively charged for pH values lower than the isoelectric point of arginine (pI = 10.75) and thus are adsorbed on the negatively charged *c* plane of the HAP crystal, which is rich in phosphate and hydroxide ions. On the contrary, L-arginine molecules are negatively charged at pH values greater than pI, so they interact with the calcium ions of the *a* plane of the HAP crystal, which is positively charged. As a result, growth of HAP crystals with plate-like and rod-like morphologies occurs, respectively [28,29].

In our case, the pH value of the original arginine solution gradually decreases and is stabilized at around 10.25, during dropwise addition of the reactants. However, needle-like HAP crystals are indeed formed for the 2S series’s samples due to the use of Tris salt solution as a solvent instead of distilled water. In detail, during synthesis of the 2S series’s suspensions, Tris buffer is used as a solvent for the L-arginine precursor solution (the solvent of the CeO_2_ suspension is Tris salt solution). The hydroxyl groups (OH^−^) present in the crystal of Tris salt interact with the functional amino groups (–NH_3_^+^) of the arginine molecules and thus, Tris-L-arginine complexes are formed, before the synthesis of hydroxyapatite even begins. Consequently, calcium ions (Ca^2+^) interact with the remaining negatively charged carboxyl group (–COO^−^) of L-arginine molecules and form calcium–arginine complexes on the calcium rich planes of the HAP crystal. The crystal plane that is transverse to the c-axis of the HAP crystal acts as a spot for further nucleation of the HAP phase and in this way, growth of needle-like HAP crystals is achieved.

During synthesis of the 3S series’s suspensions, Tris buffer is additionally used as a solvent for the Ca(OH)_2_ precursor suspension. The calcium ions in the precursor suspension complex with the Tris ions [30] before they even enter in the arginine solution. In this way, the amount of the remaining Ca^2+^ ions that are available for HAP formation in the final suspension is reduced, while the L-arginine–Tris complexation mechanism continues at the same time, as described above. Thus, both needle-like (Figure 2i) and also rod-like HAP crystals are obtained, which coalesce in larger aggregates (Figure 2h). TEM images of the 1S—4% CeO_2_ scaffold (Figure 2a,b) reveal that HAP synthesis has taken place at pH values lower than the isoelectric point of L-arginine, resulting in the formation of spherical HAP nanoparticles. More specifically, the fact that only spherical shaped HAP is observed in the corresponding TEM images is due to the fact that oriented growth of the HAP crystals was performed to a different direction than that of the crystals of the 2S-4% CeCO_2_ scaffold. The pH drop led the growth of HAP crystals to take place mainly in the direction of the a and b axes, hence the more spherical crystal formations. Furthermore, other calcium phosphate phases were crystallized in addition to HAP due to the drop in pH. This could explain why HAP phase was not identified during XRD analysis. Excess Ca^2+^ could have formed the observed spherical CaCO_3_ crystals.

### 3.3. XRD Analysis

**Figure 3:**
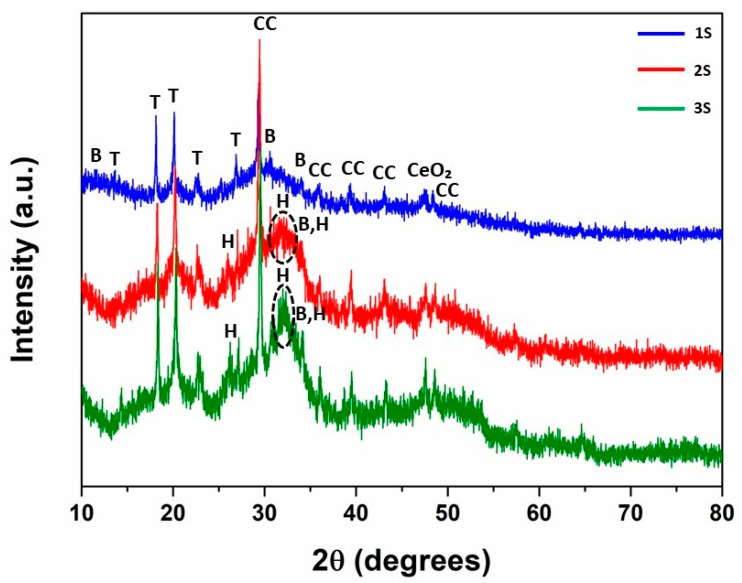
XRD spectra of the 1S—4% CeO_2_, 2S—4% CeO_2_, and 3S—4% CeO_2_ HAP-CeO_2_ composite scaffolds. The identified phases are: **B**: CaHPO_4_·2H_2_O (PDF No. 09-0077), **H**: Ca_5_(PO_4_)_3_(OH) (PDF No. 09-0432) (dashed line circular indications), **T**: C_4_H_11_NO_3_ (PDF No. 33-1699), **CC**: CaCO_3_ (PDF No. 05-0586), **CeO_2_**: CeO_2_ (PDF No. 34-0394).

The acquired XRD spectra of the HAP-CeO_2_ scaffolds are presented in Figure 3. The phase of brushite (JCPDS No. 09-0077) was detected for the 1S—4% CeO_2_ scaffold at 2θ values of 11.71°, 30.57°, and 34.08°, which correlate to the respective main (hkl) indices—(020), (−221), and (−220), respectively. Although synthesis of the 1S series’s suspension is performed in an L-arginine environment (pH ≈ 11), a local decrease in pH and subsequent aging at low temperatures (<50 °C) might lead to the formation of a brushite phase that fails to transform into stoichiometric HAP [31]. Additionally, calcium-deficient hydroxyapatite crystals are formed due to lower local pH values, which result in lower crystallinity of the formed HAP phase [32]. Therefore, the HAP phase is not detected in the sample due to its nanostructure [33], contrary to the detection of phases of larger aggregated crystals. This observation is in agreement with the results of EDS analyses during SEM characterization of the scaffolds (Figure S4). The crystallization of HAP is confirmed for the examined scaffolds of the 2S and 3S series. The HAP phase (JCPDS No. 09-0432) is detected at 2θ values of 25.90°, 31.75°, 32.15°, 33.04°, and 33.92° (Figure 3, dashed line circular indications), which are attributed to the respective crystal planes indicated as (002), (211), (112), (300), and (202), respectively.

Additionally, the main and minor peaks of the calcite phase (JCPDS No. 05-0586) are matched at 2θ values of 29.43°, 36.01°, 39.46°, 43.06°, and 48.60° for the respective crystallographic planes (104), (110), (113), (202), and (116). Among the formed peaks, the one at 29.43° overlaps the peak at 29.27°, which is assigned to the brushite phase. The detection of calcite phase also explains the formation of spherical crystallites that were observed during TEM examination of the 1S series’s sample (Figure 2a–c). Nucleation of the calcite phase in an aqueous environment starts from the precursor phase of amorphous calcium carbonate (ACC) after a moderate level of supersaturation. Thus, metastable compounds (e.g., proto-calcite) are formed, which are characterized by low crystallinity and spherical microstructure [34]. Among the aforementioned phases, the stabilization of those with higher crystallinity degree is enhanced by the presence of bio-macromolecules under ambient conditions [35]. This is the reason why the detected calcite phase is characterized by elevated crystallinity compared to other metastable phases, though it is comprised of spherical crystallites instead of the typical calcite crystals of cubic microstructure [36].

Moreover, traces of Tris salt phase (JCPDS No. 33-1699) are detected at 2θ angles of 14.35°, 18.13°, 20.22°, 22.61° and 26.86° relative to the (110), (111), (200), (210) and (112) planes. The peak at 20.22° overlaps the respective peaks of the brushite phase (≈20°). Finally, a secondary peak of CeO_2_ (JCPDS No. 34-0394) is discerned at a 2θ of 47.49° for the (220) plane in the acquired spectra. Overall, the moderate degree of crystallization of the detected phases in the examined spectra stipulates the existence of said phases at the nanoscale.

### 3.4. FTIR Spectroscopy

**Figure 4:**
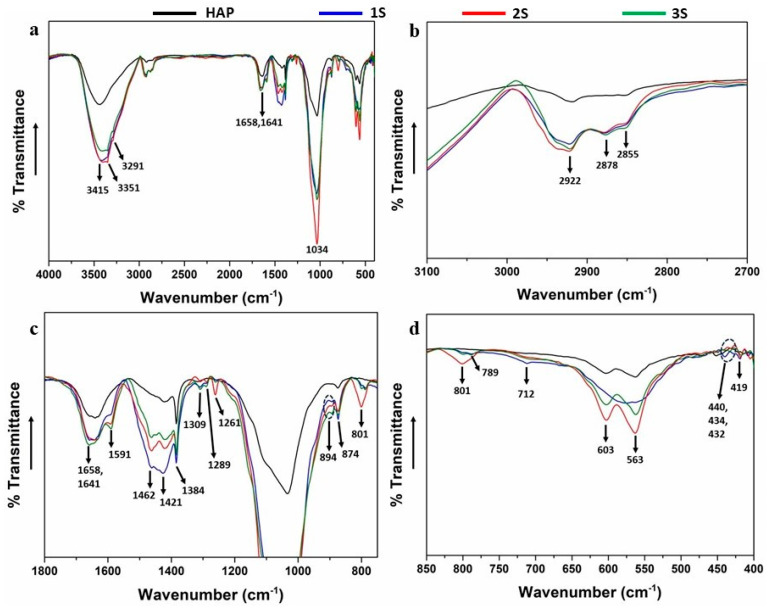
(**a**) FTIR spectra of the 1S—4% CeO_2_ (blue line), 2S—4% CeO_2_ (red line) and 3S—4% CeO_2_ (green line) HAP-CeO_2_ composite scaffolds and of HAP reference scaffold (black line). Magnified regions of interest (**b**) 3100–2700 cm^−1^, (**c**) 1800–750 cm^−1^, and (**d**) 850–400 cm^−1^ from the original FTIR spectra (4000–400 cm^−1^) are presented separately.

As is known, the characteristic peak at the FTIR spectrum for liquid water (H_2_O) are shown in the 3500–3200 cm^−1^ broad band, which refers to the O–H bond stretching vibration and which is accompanied by a narrower peak at around 1630 cm^−1^, corresponding to bending of the same bond [37]. Regarding the FT-IR spectra of the HAP-CeO_2_ scaffolds (Figure 4a), a broad band appears at 3415 cm^−1^, and smaller peaks are discerned at 3351 cm^−1^ and 3291 cm^−1^. Additionally, characteristic bending vibration peaks of the O–H bond are located at 1658 cm^−1^ and 1641 cm^−1^ [28]. The appearance of more than one different peak in the characteristic regions of O–H bond implies that hydroxyl groups (OH^−^) present in the samples may be associated with complex formations, either belong to water hydration molecules that have penetrated into the lattice of the examined material or belong to the adsorbed water molecules on the surface of the material. Therefore, multiple peaks referring to the O–H bond confirm the creation of hydrogen bonds between organic-inorganic substances [37]. The small peak at 2922 cm^−1^ and the two nearby small peaks at 2878 cm^−1^ and 2855 cm^−1^ are attributed to the asymmetric and symmetric stretching of the C–H bond of the (–CH_2_–) group that is contained in chitosan and L-arginine biopolymers (Figure 4b).

The detected peak at 1591 cm^−1^ refers to scissor vibration of the N–H bond of primary amino group (–NH_2_), which is present in the molecules of both chitosan and L-arginine biopolymers, as well as in the Tris molecule. However, no corresponding peak of the N–H bond appears at the FTIR spectrum of the HAP reference sample, nor at the FTIR spectrum of Tris [38]. Therefore, it is concluded that both the peak at 1591 cm^−1^ and the adjacent peak at 1462 cm^−1^, result from the substitution of (PO_4_)^3−^ groups in the HAP crystal and the formation of Ca^2+^-Tris complexes. The peak at 1421 cm^−1^ and the sharper peak at 1384 cm^−1^ represent the C–O bond stretching vibrations of the carboxyl group (–COOH) [37,39], present in the molecules of L-arginine [40] or chitosan [41], respectively. However, since composite materials are examined, it is possible that some bonds’ vibrations might overlap. Specifically, the peak at 1421 cm^−1^ along with the small peaks at 874 cm^−1^ and 712 cm^−1^ could be attributed to the stretching vibration and the out-of-plane bending vibration of the C–O bond for the (–CO_3_)^2−^ group [42,43], which indicates the formation of CaCO_3_ compound in the final suspension during synthesis [44]. The fact that these peaks are protruded at the FTIR spectrum of the 1S series’s sample is in agreement with the XRD and TEM findings for the specific sample. It is observed that the peaks at 1309 cm^−1^, 1289 cm^−1^, 1261 cm^−1^, and 894 cm^−1^ (Figure 4c) appear only at the FTIR spectra of the HAP-CeO_2_ samples but not in the respective spectra of the HAP reference scaffold. According to the literature [38,45,46], these peaks indicate the presence of Tris and overlap the corresponding twisting and rocking vibrations that are traced in a similar HAP/L-argHCl nanocomposite system [47,48].

The large band at 1034 cm^−1^ (Figure 4a), along with the two smaller peaks at 603 cm^−1^ and 563 cm^−1^ (Figure 4d), constitute the characteristic major and minor peaks, respectively, of the HAP phase at the FTIR spectra [49]. The main peak represents the stretching vibration and the two minor peaks the bending vibrations of phosphate groups (PO_4_)^3−^ [37]. The broad band at 1034 cm^−1^ overlaps the weaker vibrations of biopolymers, such as the stretching vibration of the C–N bond attributed to the primary amino group (–NH_2_) that is present in the L-arginine molecule or the free primary amino group (–NH_2_) in C_2_ position of the chitosan monomer [50]. The peaks at 801 cm^−1^ and 789 cm^−1^ (Figure 4d) refer to the out-of-plane bending vibration N–H of the primary amino group (–NH_2_) that exists in the molecules of both chitosan and L-arginine biopolymers, respectively, and also in the Tris molecule. It is worth noticing that the peaks at 1261 cm^−1^, 1034 cm^−1^, 801 cm^−1^, 603 cm^−1^ and 563 cm^−1^ are particularly protruded at the FTIR spectrum of 2S series’s sample compared to the corresponding spectra of the 1S and 3S series’ samples. This supports the synthesis mechanism that was discussed above, regarding the formation of Tris-L-arginine complexes during the synthesis of HAP suspensions and also, assisted growth of needle-like HAP crystals in a Tris environment. Finally, the peaks that are formed at 440 cm^−1^, 434 cm^−1^, 432 cm^−1^ [17,51,52], and 419 cm^−1^ [53] are characteristic of the Ce–O bond vibration and confirm the presence of CeO_2_ in the samples of the 1S, 2S, and 3S series. The assignment of peaks in the FTIR spectra of the HAP-CeO_2_ scaffolds of series 1S, 2S, 3S and the reference HAP scaffold are tabulated below (Table 2).

### 3.5. DMA

**Figure 5:**
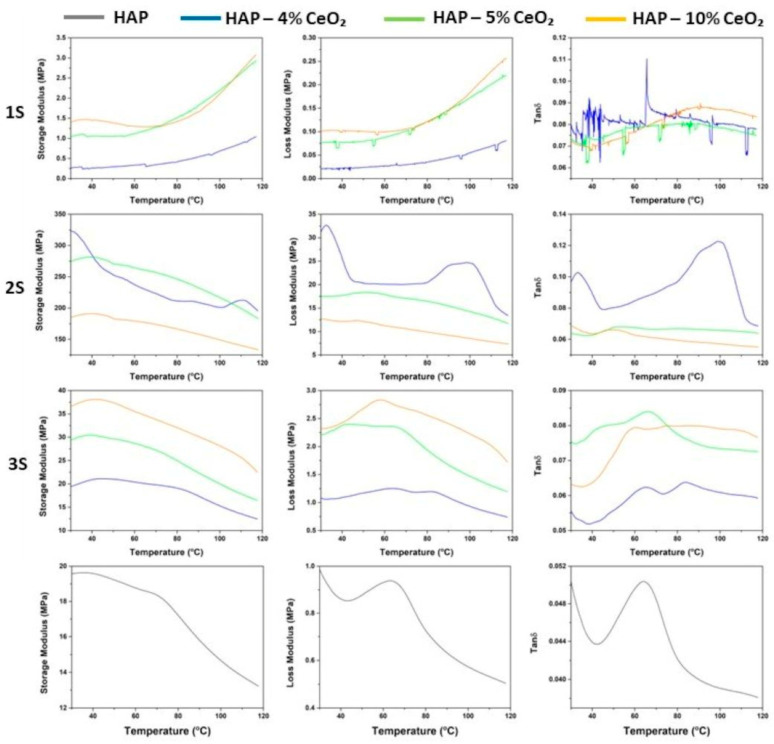
Storage modulus E′ (MPa), loss modulus E″ (MPa), and tanδ graphs of the 1S, 2S, and 3S HAP-CeO_2_ composite scaffolds and of the HAP reference scaffold.

From the following DMA graphs (Figure 5), it is observed that the mechanical response of the 2S and 3S series’ HAP-CeO_2_ scaffolds to the applied load is that of a typical semicrystalline material. In addition, the specific scaffolds show the greatest similarity to the HAP reference sample, regarding their viscoelastic behavior. In the tanδ graph of the HAP scaffold, the peak at 65 °C represents the glass transition temperature (T_g_) of chitosan [54], beyond which both its storage and loss moduli are decreased at a relatively fast rate. This is justified, since increasing temperature negatively affects the rigidity of the scaffolds, and for temperature values above T_g_, their ability to store energy is greatly impaired.

The 2S series’s scaffolds are characterized by highly crystalline, since the recorded values of storage modulus increased by one order of magnitude compared to the corresponding values of the 3S series’s scaffolds and by two orders of magnitude compared to the corresponding values of the 1S series’s scaffolds. Among the 2S series’s scaffolds, 2S—10% CeO_2_ and 2S—4% CeO_2_ scaffolds exhibit the lowest and the highest degree of crystallinity, respectively. According to the literature [55], as crystallinity increases, it is possible that the Tg moves to a region of lower temperatures. This is confirmed, as the T_g_ of chitosan is moved to a lower temperature of about 50 °C for the 2S—5% CeO_2_ and 2S—10% CeO_2_ scaffolds and to 40 °C for the 2S—4% CeO_2_ scaffold. Particularly, another intense peak is distinguished in the tanδ graph of the 2S—4% CeO_2_ scaffold at around 90–100 °C. It is mentioned that simultaneous temperature and force load can trigger orientation and/or crystallization phenomena even during glass transition of the material [56], even for the small stresses (strain) applied during DMA analysis. Therefore, the detected peak at 90–100 °C implies the occurrence of crystallization phenomena [57] (Figure S5). This phenomenon is also known as cold crystallization and is representative of how the material responds when deformation is imposed on the material, due to an increase in temperature. In this way, thermal stability of the material is achieved at specific temperatures. Beyond the cold crystallization temperature (T_c_), the 2S—4% CeO_2_ scaffold enters a second rubbery plateau region (E′ (MPa)-T (°C) graph). It is worth mentioning that between temperatures T_g_ and T_c_ in the E″ (MPa)-T (°C) graph, the curve of the loss modulus is a straight line, which is an indication that the scaffold does not undergo dimensional shrinkage during DMA experiment [56].

T_g_ of chitosan is discerned 65 °C for the 3S series’s scaffolds. Additionally, the occurrence of cold crystallization phenomena is detected at about 85 °C for 3S—4% CeO_2_ and 3S—10% CeO_2_ scaffolds (2nd peak in the respective tanδ graphs). It is mentioned that, T_g_ and T_c_ peaks are not always distinct and may appear a single temperature region, as presented in the tanδ graph of 3S—10% CeO_2_ scaffold. The smaller peak at around 50 °C in the tanδ graph of 3S—5% CeO_2_ scaffold is attributed to a sub-T_g_ relaxation effect and is likely the result of chitosan interaction with polyethylene glycol. The very low values of storage modulus that are recorded for the 1S series’s scaffolds are an indication of a low degree of crystallinity of the samples. Moreover, the storage moduli of the scaffolds remain almost constant until they present an increasing trend during the upper values in the examined temperature range. This kind of response implies an increase in viscosity due to the formation of intermolecular hydrogen bonds and therefore an increase in the stiffness of the scaffolds [58]. Increased stiffness intercepts the movement of polymeric chains, which is the reason why no distinguishable sharp peak is formed in the T_g_ region in the tanδ graphs of the specific scaffolds.

### 3.6. UCT and Cyclic Loading

All the scaffolds, and also the reference sample, behave as thermoplastic polymers since their respective stress–strain curves show characteristic regions, such as the linear elastic region, the plateau stage, and the densification stage [59]. Transition from the linear elastic region to the plateau stage is performed smoothly for most samples. However, the buckling region is clearly visible in the curves of samples 2S—4% CeO_2_, 3S—5% CeO_2_ and less visible in the curves of samples 2S—10% CeO_2_ and 3S—10% CeO_2_, which also signals the end of the linear elastic region. Additionally, scaffolds 2S—4% CeO_2_ and 3S—5% CeO_2_ are the ones that show the highest compressive strength. This is due to the development of needle-like HAP nanocrystals in their microstructure, which were observed with TEM. The HAP nanocrystals align in the direction of ice crystal growth during the freezing stage of the lyophilization process, which coincides with the axis of mechanical loading. As a result, scaffolds comprised of needle-shaped HAP crystals present increased resistance when subjected to mechanical loading, compared to that exhibited by scaffolds comprised of spherical HAP nanocrystals, such as the scaffolds of the 1S series. Apart from the HAP particle morphology, the percentage of the reinforcing phase can largely impact the mechanical properties of the scaffolds [19]. In the present work, higher percentage of the reinforcing CeO_2_ phase does not seem to enhance the mechanical response of the scaffolds, since HAP—10% CeO_2_ samples show the lowest compressive strengths. The presence of calcium carbonate, which is characterized by reduced compressive strength compared to HAP [60], negatively impacts the mechanical strength of the 1S scaffolds, which show the lowest compressive strength among the examined scaffolds.

**Figure 6:**
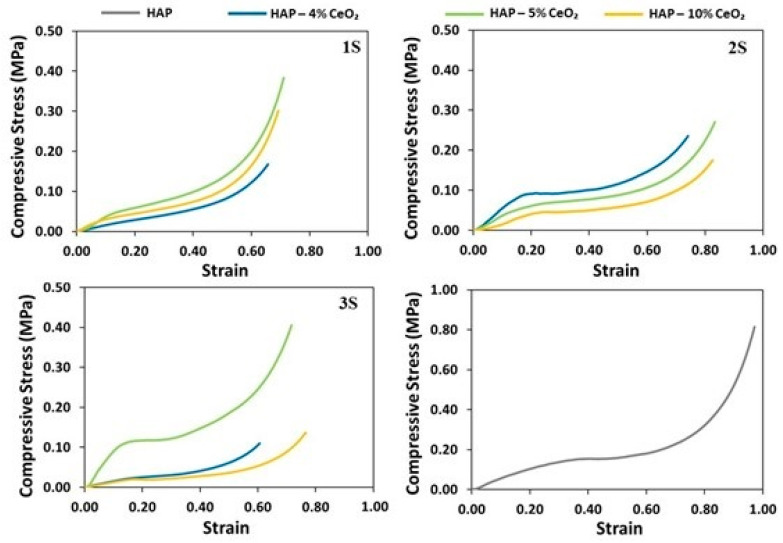
Stress (MPa)–strain curves under uniaxial compression for the 1S, 2S, and 3S HAP-CeO_2_ composite scaffolds and for the HAP reference scaffold.

According to Table 3, scaffolds 2S—4% CeO_2_ and 3S—5% CeO_2_ are characterized by the greatest average values of compressive moduli, equal to 0.59 MPa and 0.86 MPa, respectively. These values are comparable to the average compressive modulus of the HAP reference scaffolds, which is equal to 0.62 MPa. The ability of porous foams to absorb energy is related to the extent of the plateau region [61]. Therefore, improvement of a material in terms of its ability to absorb energy can be carried out by improving the material itself and also by achieving greater deformation at the onset of densification of the porous structure (onset of densification is moved to the right, along the strain axis). The 2S—4% CeO_2_ and 3S—5% CeO_2_ scaffolds show the highest average values of energy absorption per unit of volume. Thus, they need to absorb larger amounts of energy before starting to degrade and passing into the plateau region (Figure S6). In addition, the plateau area of the specific scaffolds increases due to an increase in their modulus of elasticity in compression. The remaining scaffolds, as well as the reference scaffold despite its relatively increased compressive modulus, pass almost directly from the linear elastic region to the plateau region and begin to plastically deform almost instantaneously (Figure 6). Thus, they show an elongated region of plastic deformation before entering into the densification stage. Therefore, energy absorption for the 2S—4% CeO_2_ and 3S—5% CeO_2_ scaffolds is related to strengthening of their construction material, while energy absorption for the rest of the scaffolds is related to the increased plastic deformation area.

**Figure 7:**
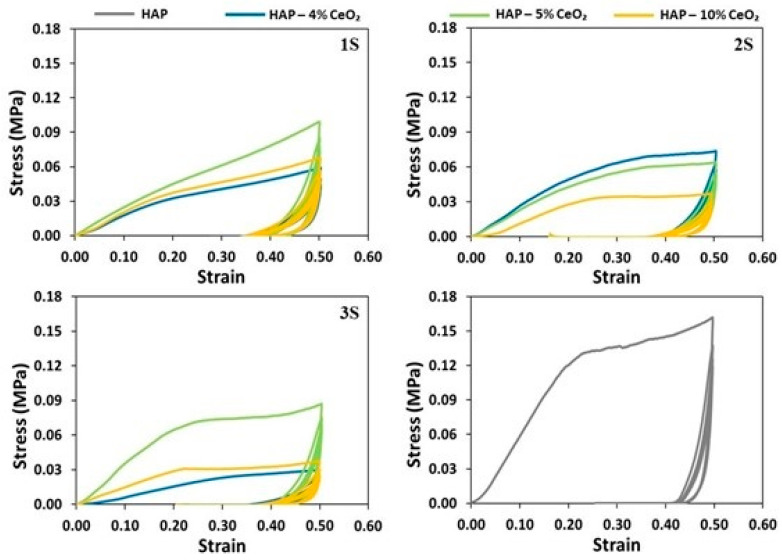
Stress (MPa)–strain curves during compressive cyclic loading up to 50% strain for 5 cycles for the 1S, 2S, and 3S HAP-CeO_2_ composite scaffolds and for the HAP reference scaffold.

From the loading–unloading curves of the examined samples (Figure 7), it is observed that the scaffolds of the 1S series, which presented the lowest average values of compressive moduli, also present the highest elastic recovery per cycle (Tables S1 and S2). Nevertheless, all of the tested scaffolds seem to follow a similar pattern of degradation. Specifically, upon completion of the first two loading-unloading cycles the scaffolds are plastically deformed to a great extent. As a result, upon completion of the third cycle and up to the fifth cycle, the additional energy required for plastic deformation is comparatively significantly less.

### 3.7. Scaffold Porosity Measurements

**Figure 8:**
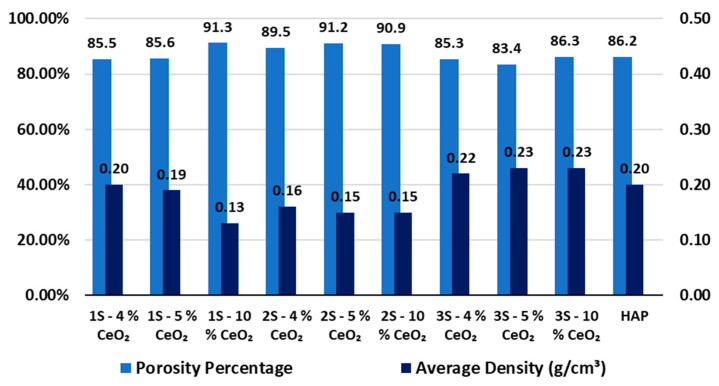
Average values of porosity percentage (%) and density (g/cm^3^) of the 1S, 2S, and 3S series’ HAP-CeO_2_ composite scaffolds and the reference HAP scaffold, obtained using the Archimedes method.

As it is observed (Figure 8), the average open porosity content obtained through the Archimedes method is calculated to be greater than 80%. Therefore, the development of an interconnected porous network is confirmed, since the solvent manages to penetrate inside the scaffolds. The Archimedes method enables porosity calculation from data (Table S3) acquired from the total volume of three-dimensional structures, which also explains the high porosity percentages that are calculated. Although excessive content of internal porosity can impair the mechanical properties of the scaffolds, in this case, it is suggested that the microstructure of the scaffold material primarily affects their mechanical response. Regarding the average values of the scaffolds’ density (Figure 8), they follow a reverse profile compared to the one of the scaffolds’ porosity contents, as expected. However, since open porosity of all the samples consists of very close average values, the lower calculated density of the 2S series’s scaffolds could be an indication of their denser microstructure.

### 3.8. Swelling, Absorption and Dissolution Assessment

**Figure 9:**
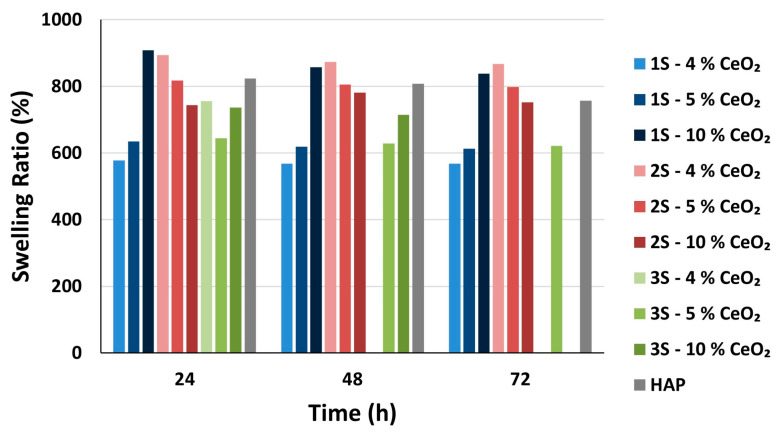
Swelling ratio SR_i_ (%) of the HAP-CeO_2_ composite scaffolds and of HAP reference scaffold, after immersion into PBS solution (pH = 7.4, @37 °C) for i hours (i = 24, 48, 72).

The ability of a scaffold to retain fluids in its pore system enables the influx of cell nutrients into its interior [62] and therefore dictates adhesion, growth and differentiation of the cells [63]. In this way, the swelling ability of a scaffold constitutes an important indicator of its potential function and effectiveness within the human body. From the swelling studies performed on the HAP-CeO_2_ scaffolds, it is shown that the swelling ability of the scaffolds is not affected by the amount of CeO_2_ introduced into the scaffolds, since the average swelling ratios differ among scaffolds of different series but with the same CeO_2_ content (Figure 9). It is also evident that the set of average values of swelling ratios follow a downward trend, which is expected as a state of equilibrium is reached during immersion of the scaffolds into the PBS, over time. The scaffolds approach the equilibrium state with the surrounding liquid, as early as the first 24 h of immersion into the PBS (Table S4). The 3S—4% CeO_2_ and 3S—10% CeO_2_ scaffolds do not stabilize inside the buffer solution and are dissolved after 48 and 72 h, respectively. As has already been mentioned, the 3S series’s scaffolds are most likely to contain a lower percentage of HAP phase due to their synthesis process, which leads to their increased solubility in the buffer solution. However, 3S—5% CeO_2_ samples present close average values of swelling ratios even after 72 h, possibly due to their different internal microstructure (Figure 1h), which seems to have a greater effect on their swelling ability compared to their composition. A diagram of the swelling ratios (%) of the scaffolds as a result of liquid absorption through their porous network is presented below (Figure 9).

**Figure 10:**
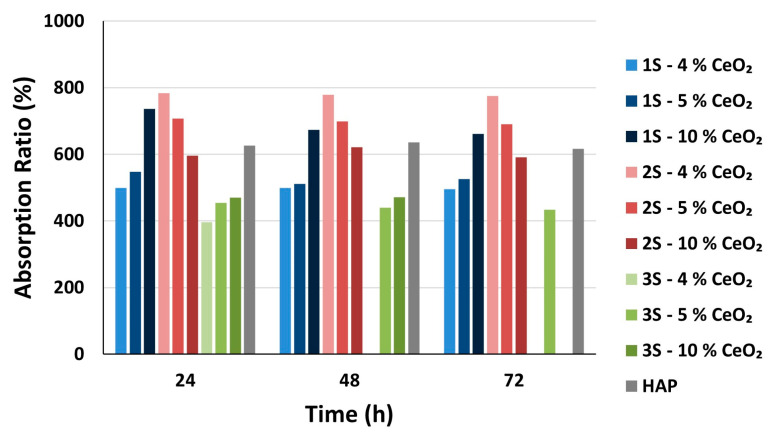
Absorption ratio AR_i_ (%) of the HAP-CeO_2_ composite scaffolds and of HAP reference scaffold, after immersion into PBS solution (pH = 7.4, @37 °C) for i hours (i = 24, 48, 72).

After observation of Figure 9 and Figure 10, it is evident that the ability of the scaffold to absorb liquid with its porous network is superior to the absorption ability of the scaffold material. The average values of absorption ratio have already been stabilized after 24 h and are retained almost the same after 72 h (Table S5). It is noted that average absorption ratio values (AR_72_) and average swelling ratio values (SR_72_) after 72 h of immersion into PBS, show a greater discrepancy amongst them than the average absorption ratio values (AR_72_) after 72 h of immersion into PBS and average swelling ratio values (SR_5_) after 5 min of immersion into isopropanol (Table S6). This implies that in a wet environment, the scaffold material quickly absorbs the maximum amount of liquid and that subsequent absorption continues through the pore system of the scaffold. This is considered to be a desirable feature, as excessive swelling can lead to collapse of the three-dimensional scaffold network and, consequently, impairment of the mechanical strength of the scaffolds.

**Figure 11:**
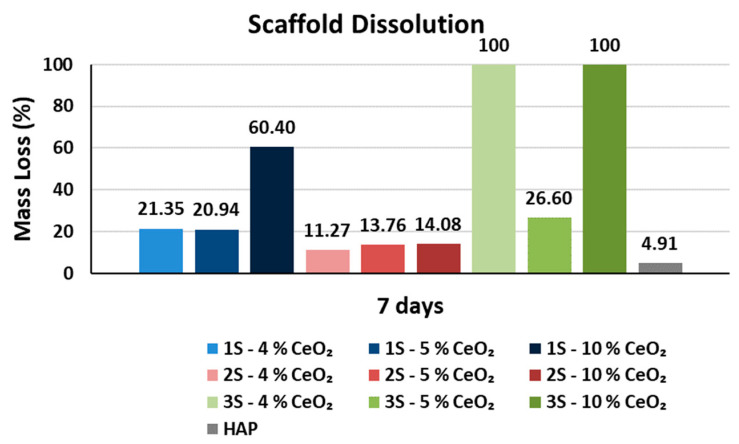
Mass loss ratio D (%) of the HAP-CeO_2_ composite scaffolds and of HAP reference scaffold, after immersion into PBS solution (pH = 7.4, @37 °C) for 7 days.

Regarding scaffold dissolution, the 2S series’s scaffolds undergo significantly less mass loss compared to the rest of the HAP-CeO_2_ scaffolds (Figure 11, Table S7). Moreover, the amount of integrated CeO_2_ does not affect the scaffold’s solubility in the liquid medium in general. The 2S series’s scaffolds are characterized by the lowest average values of density and also the highest porosity content (Figure 8). At the same time, these scaffolds show great stability in the liquid medium over time, despite their “open” structure. Therefore, it is safe to assume that their dissolution behavior in the PBS is due to their microstructure as well as their composition, which is characterized by a mineralized phase of HAP nanoparticles strongly cross-linked with the available biopolymers [64].

### 3.9. Antifungal Study on CeO_2_ Suspension

From the antifungal study on the CeO_2_ suspension it is concluded that CeO_2_ nanoparticles show moderate antifungal activity. Although, they present no antifungal activity at the lowest studied concentrations (≤15.8 ppm), their antifungal properties were evident at concentrations of nanoparticles higher than 86.1 ppm against both *Aspergillus flavus* and *Aspergillus fumigatus* (Table 4). Thus, it can be reported that the incorporation of CeO_2_ suspension into hydroxyapatite scaffolds is expected to improve the overall biological properties of the final HAP-CeO_2_ scaffolds.

## 4. Conclusions

Three different series of the composite HAP-CeO_2_ scaffolds were prepared. Their examination and characterization showed that the samples of the 2S series present the greater enhancement regarding their viscoelastic properties. However, the effect of CeO_2_’s addition to the HAP suspension is more obvious when the respective scaffolds are tested under dynamic compression conditions than in standard compression tests. It is also concluded that the samples of the 2S series present the best mechanical response—specifically, the sample with a reinforcement phase percentage equal to 4%—and that increasing the added percentage of the reinforcing CeO_2_ phase does not seem to enhance the mechanical response of the scaffolds. This is due to the growth of needle-like HAP crystals that, when oriented perpendicular to the direction of stress application, enhance the resistance of the sample to deformation. The orientation of the particles in a specific direction is achieved through the lyophilization technique used to shape the ceramic paste. The morphology of the HAP nanocrystals in the final composite scaffold is a result of oriented growth which is determined by the presence of excess Tris salt in the arginine solution. The hydroxyl groups (OH^−^) present in the Tris salt crystal interact with the functional amino groups (–NH_3_^+^) in the arginine molecule. Thus, Tris–L-arginine complexes are formed before the synthesis of hydroxyapatite even begins. Therefore, calcium ions (Ca^2+^) interact with the remaining negatively charged carboxyl groups (–COO^−^) on the L-arginine molecules and form calcium–arginine complexes on the positively charged planes of the HAP crystal, which are transverse to axes a and b. The crystal plane that is transverse to the c-axis of the HAP crystal is an available point for further nucleation of the HAP phase and in this way the growth of acicular HAP crystals is achieved.

The pores of the optimal scaffolds had an average size equal to 100 μm and the percentage of open porosity content was equal to a minimum of 89.5%, as calculated from measurements made by the Archimedes method. In addition, the 2S series’s samples showed the lowest dissolution rate in phosphate buffered saline (PBS), overall, although it is greater than the dissolution rate of the HAP control sample. It was also found that the amount of added CeO_2_ phase does not affect the solubility of the scaffold in the liquid medium. The slow dissolution rate of the 2S series’s scaffolds in PBS is due to their particular microstructure as well as their composition, which consists of the HAP nanoparticle phase strongly cross-linked with available biopolymers. Finally, after antifungal studies performed at the CeO_2_ suspension, it was found that the ceria nanoparticles present antifungal behavior against both *Aspergillus flavus* and *Aspergillus fumigatus* species at concentrations higher than 86.1 ppm. Thus, the produced composite HAP-CeO_2_ scaffolds constitute a promising antifungal bone graft material for potential biomedical applications.

## Figures and Tables

**Table 1:** Code names of the examined compositions.

CeO_2_ Content/ Series	1S	2S	3S
**4%**	1S—4% CeO_2_	2S—4% CeO_2_	3S—4% CeO_2_
**5%**	1S—5% CeO_2_	2S—5% CeO_2_	3S—5% CeO_2_
**10%**	1S—10% CeO_2_	2S—10% CeO_2_	3S—10% CeO_2_

**Table 2:** Peak assignments for the FTIR spectra of the 1S, 2S, 3S HAP-CeO_2_ composite scaffolds and of the HAP reference scaffold.

Assignment	Peak Positions (cm^−1^)
Absorbed water	3500–3200
C–H asymmetrical stretching	2922
C–H symmetrical stretching	2878, 2855
H–O–H bending	1658, 1641
Ca^2+^–Tris complexation	1591, 1462
C–O symmetrical stretching of (–COOH) group (C–O stretching of the (–CO_3_)^2−^ group)	1421, 1384 (1421)
Tris presence	1309, 1289, 1261, 894
(PO_4_)^3−^ stretching	1034
C–O out-of-plane bending of the (–CO_3_)^2−^ group	874, 712
N–H out-of-plane bending	801, 789
(PO_4_)^3−^ bending	603, 563
Ce–O	440, 434, 432, 419

**Table 3:** Average values of modulus of compression Ε (MPa) and energy absorption per unit volume EA (mJ/mm^3^) of the examined scaffolds.

Sample	E (MPa)	EA (mJ/mm³)
1S—4% CeO_2_	0.19	0.0173
1S—5% CeO_2_	0.38	0.0284
1S—10% CeO_2_	0.33	0.0241
2S—4% CeO_2_	0.59	0.0366
2S—5% CeO_2_	0.50	0.0274
2S—10% CeO_2_	0.29	0.0188
3S—4% CeO_2_	0.12	0.0105
3S—5% CeO_2_	0.86	0.0457
3S—10% CeO_2_	0.30	0.0154
HAP	0.62	0.0434

**Table 4:** Growth inhibition *Aspergillus flavus* and *Aspergillus fumigatus* treated with different concentrations of CeO_2_ nanoparticles. (+ denotes no inhibition, − denotes inhibition).

**Fungi Species**	***Aspergillus flavus***						
**Sample/Concentrations**	Control	8.6 ppm	15.8 ppm	86.1 ppm	214.1 ppm	258.2 ppm	356.3 ppm
CeO_2_ suspension	+	+	+	-	−	−	−
**Fungi Species**	***Aspergillus fumigatus***						
**Sample/Concentrations**	Control	8.6 ppm	15.8 ppm	86.1 ppm	214.1 ppm	258.2 ppm	356.3 ppm
CeO_2_ suspension	+	+	+	−	−	−	−

## Data Availability

The original contributions presented in the study are included in the article/supplementary material, further inquiries can be directed to the corresponding authors.

## Supplementary Materials

The following supporting information can be downloaded at: https://www.mdpi.com/article/10.3390/nano14131102/s1, Figure S1: SEM Images of the composite HAP–CeO_2_ scaffolds. (a–c) 1S—4% CeO_2_, (d–f) 1S—5% CeO_2_, (g–i) 1S—10% CeO_2_; Figure S2: SEM images of the composite HAP–CeO_2_ scaffolds. (a–c) 2S—4% CeO_2_, (d–f) 2S—5% CeO_2_, (g–i) 2S—10% CeO_2_; Figure S3: SEM Images of the composite HAP–CeO_2_ scaffolds. (a–c) 3S—4% CeO_2_, (d–f) 3S—5% CeO_2_, (g–i) 3S—10% CeO_2_; Figure S4: SEM images and EDS spot analyses. Detected phase of (a,b) CDHA and (c,d) HAP; Figure S5: SEM images of composite HAP–CeO_2_ scaffolds. (a) 1S—5% CeO_2_, (b,c) 1S—10% CeO_2_, (d–g) 2S—4% CeO_2_, (h) 2S—5% CeO_2_, (i) 2S—10% CeO_2_; Table S1: Modulus of compression Ε_i_ (MPa) and energy absorption per unit volume EA_i_ (mJ/mm^3^) of each of the examined HAP-CeO_2_ composite scaffolds i (i = 1, 2, 3) and of HAP reference scaffolds; Figure S6: Aggregate chart of average values of modulus of compression Ε_i_ (MPa) and energy absorption per unit volume EA_i_ (mJ/mm^3^) of each of the examined HAP-CeO_2_ composite scaffolds i (i = 1, 2, 3) and of HAP reference scaffolds; Table S2: Calculated values of plastic deformation energy per unit volume PD_i_ (mJ/mm^3^) and elastic recovery per unit volume ER_i_ (mJ/mm^3^) for each cycle i (i = 1, 2, 3, 4, 5) of each of the examined HAP-CeO_2_ composite scaffolds i (i = 1, 2, 3) and of HAP reference scaffolds; Table S3: Scaffold porosity measurements for the HAP-CeO_2_ composite scaffolds and for HAP reference scaffold acquired via the Archimedes method (used solvent: isopropanol); Table S4: Dry scaffold mass W_d_ (g), weight of swollen scaffolds W_s_ (g) and swelling ratio SR_i_ (%) of the HAP-CeO_2_ composite scaffolds and of HAP reference scaffold, after immersion into PBS solution (pH = 7.4, @37 °C) for i hours (i = 24, 48, 72); Table S5: Dry scaffold mass W_d_ (g), saturated weight W_sat_ (g) and absorption ratio AR_i_ (%) of the HAP-CeO_2_ composite scaffolds and of HAP reference scaffold, after immersion into PBS solution (pH = 7.4, @37 °C) for i hours (i = 24, 48, 72); Table S6: Average values of swelling ratio SR_i_ (%) of the HAP-CeO_2_ composite scaffolds and of HAP reference scaffold, after immersion into isopropanol for 5 min and average values of swelling ratio SR_i_ (%) and absorption ratio AR_i_ (%) after immersion into PBS solution (pH = 7.4, @37 °C) for 72 h; Table S7: Dry scaffold mass W_d_ (g), dry scaffold mass after freeze-drying W_fd_ (g) and dissolution D (%) of the HAP-CeO_2_ composite scaffolds and of HAP reference scaffold after immersion into PBS solution (pH = 7.4, @37 °C) for 7 days; High-resolution TEM images: Figure 2a–i.
