# Supplementary material for: Effect of Nanoceria Suspension Addition on the Physicochemical and Mechanical Properties of Hybrid Organic–Inorganic Hydroxyapatite Composite Scaffolds
Source: Nanomaterials (Basel). 2024 Jun 27;14(13):1102. doi: 10.3390/nano14131102 (PMC11242940; doi:10.3390/nano14131102)
Supplement: Supplementary file 1 [file nanomaterials-14-01102-s001.zip › nanomaterials-3057579-Supplementary_Information.pdf]

3.1 SEM

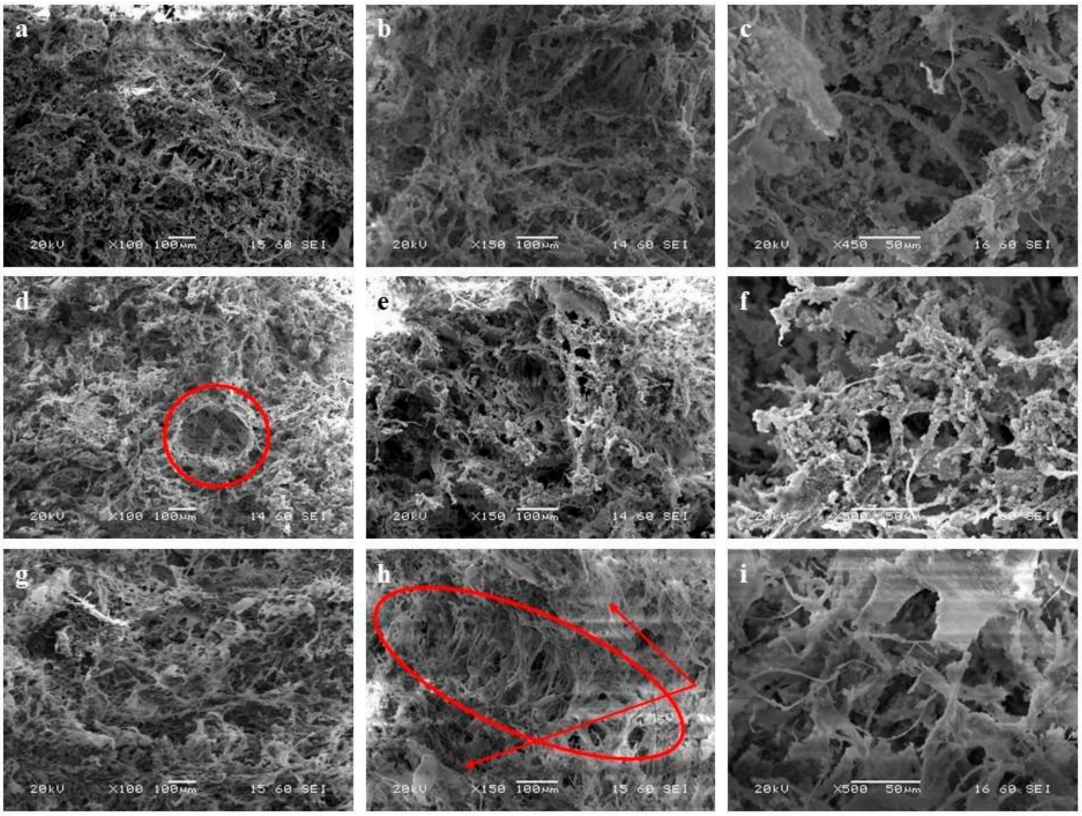

Figure S1: SEM Images of the composite HAP–CeO_2 scaffolds. (a), (b), (c) 1S – 4 % CeO_2, (d), (e), (f) 1S – 5 % CeO_2, (g), (h), (i) 1S – 10 % CeO_2.

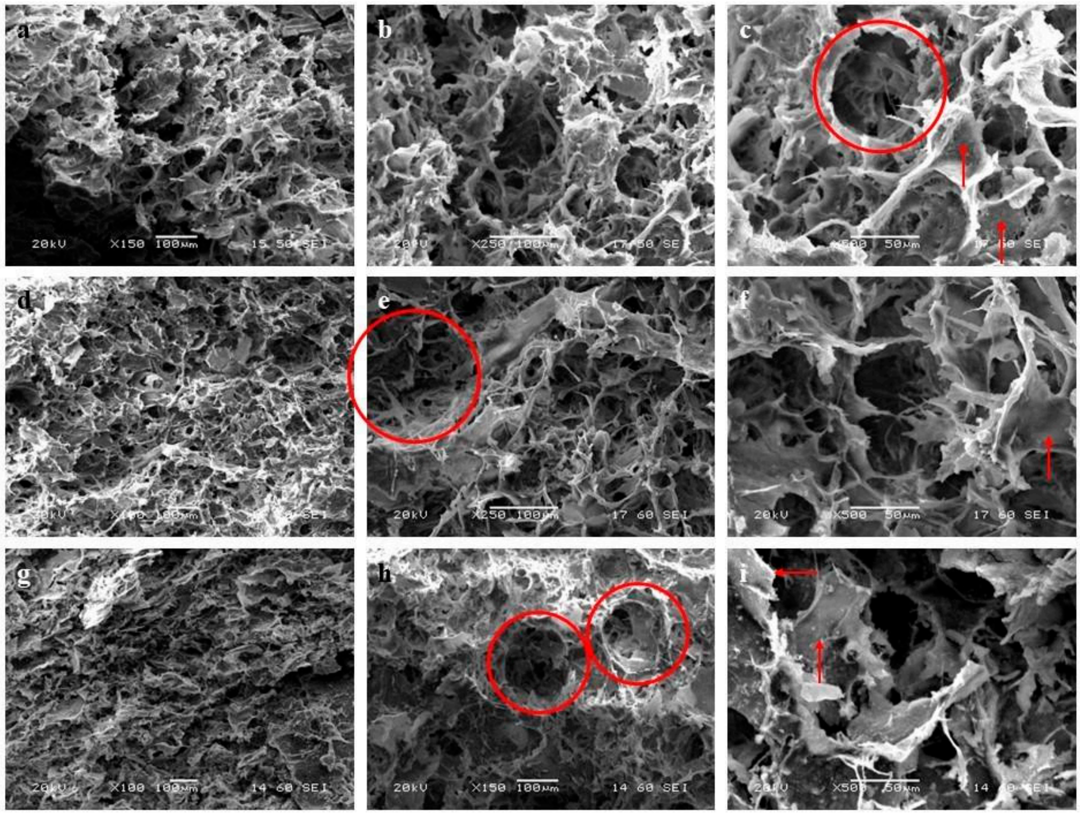

Figure S2: SEM Images of the composite HAP–CeO_2 scaffolds. (a), (b), (c) 2S – 4 % CeO_2, (d), (e), (f) 2S – 5 % CeO_2, (g), (h), (i) 2S – 10 % CeO_2.

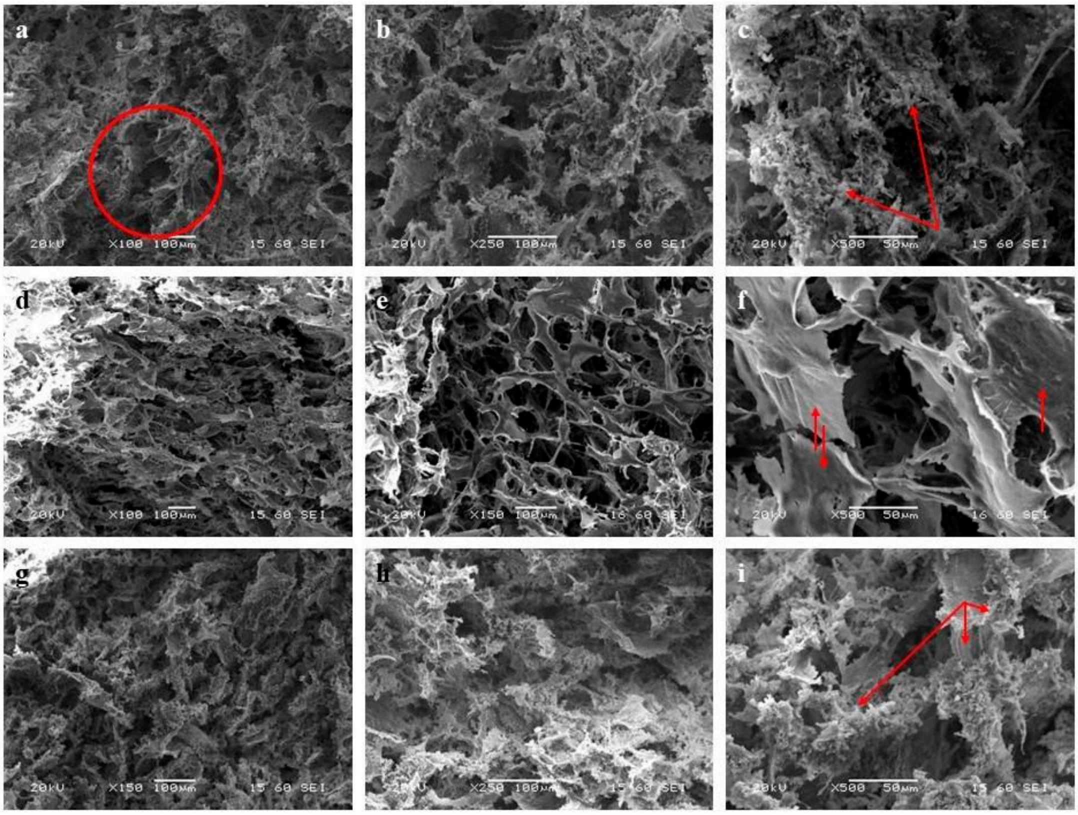

Figure S3: SEM Images of the composite HAP–CeO_2 scaffolds. (a), (b), (c) 3S – 4 % CeO_2, (d), (e), (f) 3S – 5 % CeO_2, (g), (h), (i) 3S – 10 % CeO_2.

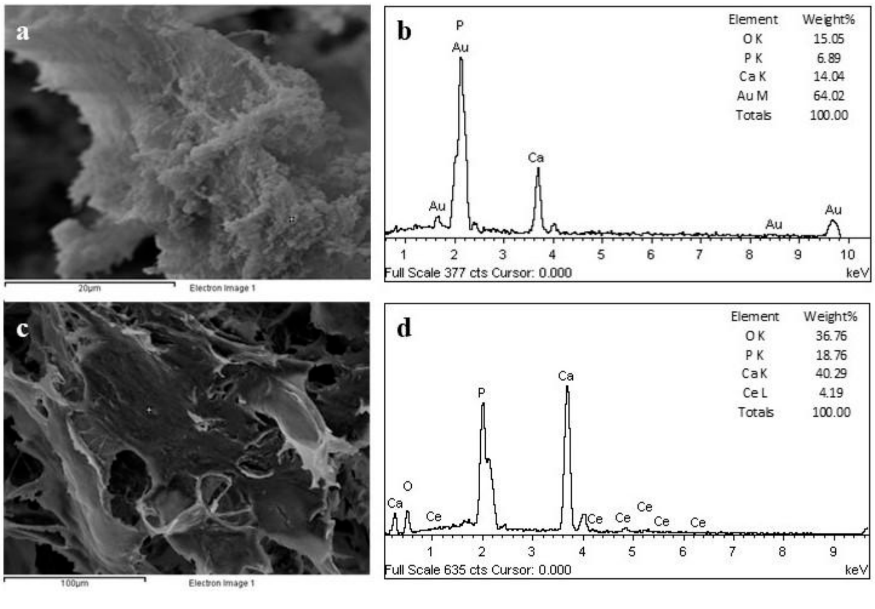

Figure S4: SEM images and EDS spot analyses. Detected phase of (a), (b) CDHA and (c), (d) HAP.

3.5 DMA

After DMA analyses the microstructural characteristics of some of the tested scaffolds are examined via SEM (Fig. S5). It is noted that the periodic growth of dense-porous zones in the 1S – 10 % CeO_2 sample appears to have been preserved even after the application of the test loads (Fig. S5b, indicated with red arrows). This microstructural feature can be beneficial as it enables the absorption of a greater amount of energy before collapse of the scaffold. Concerning the 2S – 4 % CeO_2 scaffold microstructure, it is noted that there are areas in which the pores have preserved their original shape (Fig. S5d) and others in which the pores appear to be more closed (Fig. S5e). This is due to the arrangement of the needle-like HAP crystals parallel to the load axis. As a result, the strength of the scaffold is enhanced and its original microstructure is maintained. Finally, phenomena of oriented crystallization in the direction of stress application are observed (Figs. S5f and S5g, indicated with red arrows).

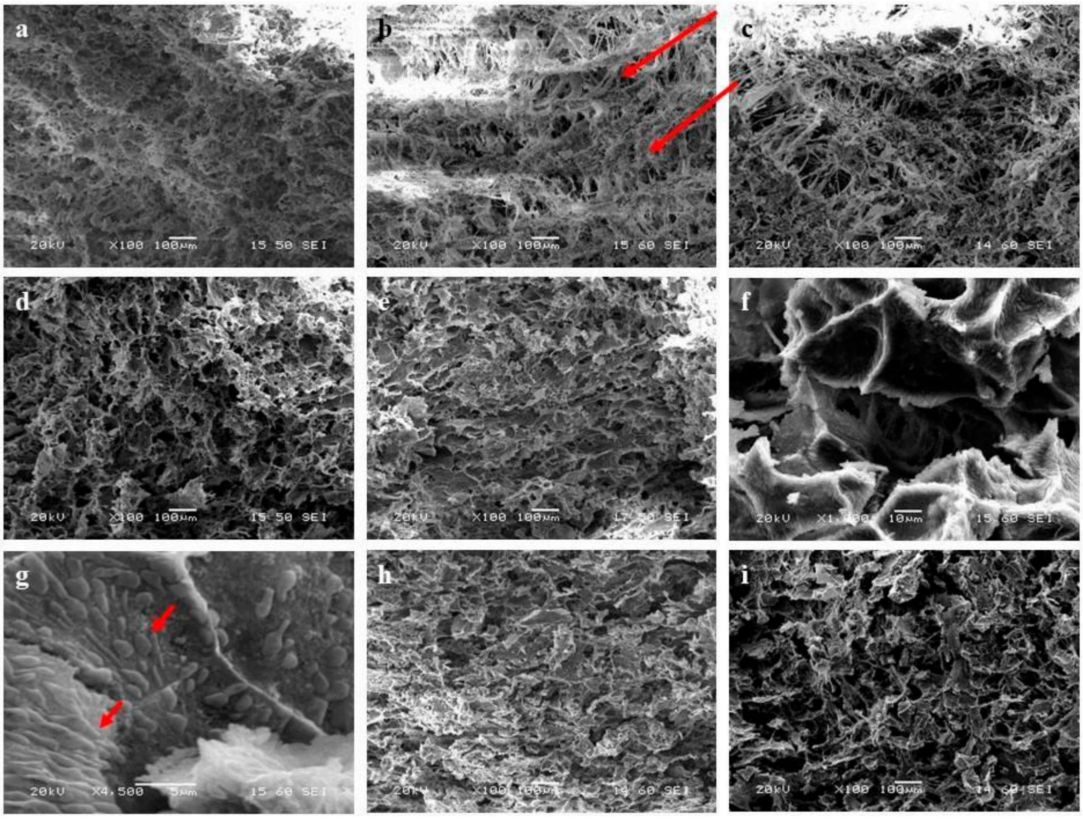

Figure S5: SEM images of composite HAP–CeO_2 scaffolds. (a) 1S – 5 % CeO_2, (b), (c) 1S – 10 % CeO_2, (d), (e), (f), (g) 2S – 4 % CeO_2, (h) 2S – 5 % CeO_2, (i) 2S – 10 % CeO_2.

3.6 UCT and cyclic loading

Table S1: Modulus of Compression E_i (MPa) and Energy Absorption per unit volume EA_i (mJ/mm^3) of each of the examined HAP-CeO_2 composite scaffolds i (i=1, 2, 3) and of HAP reference scaffolds.

	**E_i (MPa)**			**EA_i (mJ/mm^3)**		
sample	E_1	E_2	E_3	EA_1	EA_2	EA_3
1S – 4 % CeO_2	0.23	0.17	0.18	0.0171	0.0178	0.0171
1S – 5 % CeO_2	0.53	0.29	0.33	0.0333	0.0256	0.0263
1S – 10 % CeO_2	0.41	0.23	0.36	0.0257	0.0214	0.0253
2S – 4 % CeO_2	0.66	0.59	0.52	0.0336	0.0398	0.0364
2S – 5 % CeO_2	0.49	0.56	0.44	0.0246	0.0284	0.0291
2S – 10 % CeO_2	0.22	0.37	0.28	0.0197	0.0185	0.0181
3S – 4 % CeO_2	0.12	0.10	0.15	0.0106	0.0067	0.0142
3S – 5 % CeO_2	1.21	0.80	0.56	0.0569	0.0410	0.0391
3S – 10 % CeO_2	0.37	0.41	0.13	0.0152	0.0208	0.0101
HAP	0.71	0.59	0.56	0.0531	0.0391	0.0381

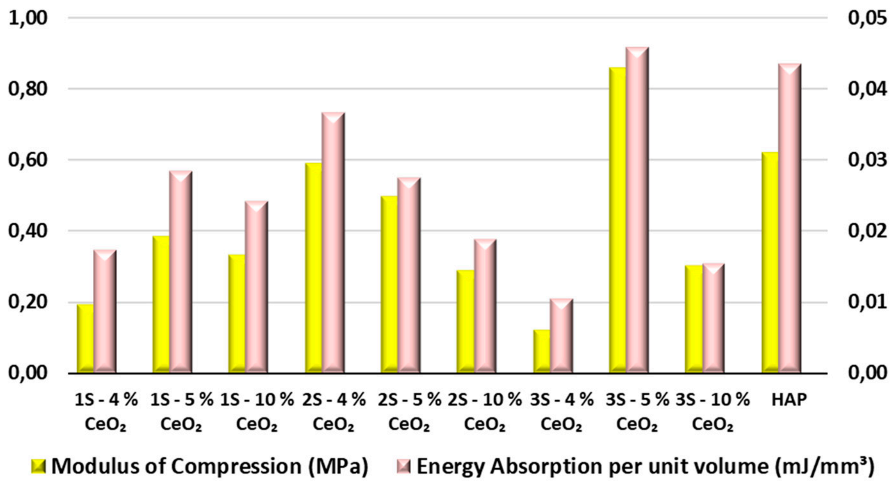

Figure S6: Aggregate chart of average values of Modulus of Compression E_i (MPa) and Energy Absorption per unit volume EA_i (mJ/mm^3) of each of the examined HAP-CeO_2 composite scaffolds i (i=1, 2, 3) and of HAP reference scaffolds.

Table S2: Calculated values of Plastic Deformation energy per unit volume PD_i (mJ/mm^3) and Elastic Recovery per unit volume ER_i (mJ/mm^3) for each cycle i (i= 1, 2, 3, 4, 5) of each of the examined HAP-CeO_2 composite scaffolds i (i=1, 2, 3) and of HAP reference scaffolds.

sample	**PD_i (mJ/mm^3) and ER_i (mJ/mm^3)**				
	PD_1 / ER_1	PD_2 / ER_2	PD_3 / ER_3	PD_4 / ER_4	PD_5 / ER_5
1S – 4 % CeO_2	0.016355	0.001709	0.001157	0.000942	0.000819
	0.001106	0.000982	0.000908	0.000854	0.000818
1S – 5 % CeO_2	0.025679	0.002399	0.001642	0.001362	0.001185
	0.001600	0.001472	0.001365	0.001310	0.001257
1S – 10 % CeO_2	0.018789	0.001965	0.001331	0.001083	0.000942
	0.001272	0.001130	0.001044	0.000982	0.000940
2S – 4 % CeO_2	0.024247	0.001572	0.001058	0.000859	0.000763
	0.001019	0.000942	0.000863	0.000817	0.000818
2S – 5 % CeO_2	0.021085	0.001367	0.000920	0.000747	0.000663
	0.000886	0.000819	0.000751	0.000710	0.000711
2S – 10 % CeO_2	0.012038	0.000906	0.000601	0.000483	0.000432
	0.000601	0.000534	0.000490	0.000513	0.000441
3S – 4 % CeO_2	0.008238	0.000800	0.000542	0.000440	0.000383
	0.000546	0.000482	0.000445	0.000421	0.000405
3S – 5 % CeO_2	0.029331	0.001847	0.001229	0.000991	0.000860
	0.001082	0.001013	0.000952	0.000910	0.000878
3S – 10 % CeO_2	0.012324	0.000806	0.000521	0.000419	0.000356
	0.000517	0.000439	0.000393	0.000365	0.000341
HAP	0.051911	0.002250	0.001432	0.001152	0.000977
	0.001628	0.001468	0.001349	0.001292	0.001247

3.7 Scaffold porosity measurements

Table S3: Scaffold porosity measurements for the HAP-CeO_2 composite scaffolds and for HAP reference scaffold acquired via the Archimedes' Method (used solvent: isopropanol).

series	CeO_2 content (%)	W_d (g)	W_{sat} (g)	W_{sus} (g)	W_{sat} - W_{sus} (cm^3)	d (g/cm^3)	P (%)	SR(%) (@5 min)
1S	4 % CeO_2	0.3242	1.6691	0.0890	1.5801	0.21	85.1%	414.84%
	4 % CeO_2	0.2967	1.6245	0.0778	1.5467	0.19	85.8%	447.52%
	5 % CeO_2	0.2942	1.6405	0.0716	1.5689	0.19	85.8%	457.61%
	5 % CeO_2	0.2825	1.6178	0.0527	1.5651	0.18	85.3%	472.67%
	10 % CeO_2	0.1671	1.3551	0.0617	1.2934	0.13	91.9%	710.95%
	10 % CeO_2	0.1763	1.3748	0.0528	1.3220	0.13	90.7%	679.81%
2S	4 % CeO_2	0.2326	1.5227	0.0738	1.4489	0.16	89.0%	554.64%
	4 % CeO_2	0.2132	1.4783	0.0703	1.4080	0.15	89.9%	593.39%
	5 % CeO_2	0.1958	1.4127	0.0696	1.3431	0.15	90.6%	621.50%
	5 % CeO_2	0.2122	1.6005	0.0884	1.5121	0.14	91.8%	654.24%
	10 % CeO_2	0.1830	1.2787	0.0665	1.2122	0.15	90.4%	598.74%
	10 % CeO_2	0.1579	1.1801	0.0608	1.1193	0.14	91.3%	647.37%
3S	4 % CeO_2	0.3383	1.6872	0.1010	1.5862	0.21	85.0%	398.73%
	4 % CeO_2	0.2144	1.0454	0.0737	0.9717	0.22	85.5%	387.59%
	5 % CeO_2	0.2893	1.3505	0.0879	1.2626	0.23	84.0%	366.82%
	5 % CeO_2	0.2946	1.3986	0.0648	1.3338	0.22	82.8%	374.75%
	10 % CeO_2	0.2904	1.4213	0.1000	1.3213	0.22	85.6%	389.43%
	10 % CeO_2	0.1199	0.5714	0.0520	0.5194	0.23	86.9%	376.56%
	HAP	0.1925	1.2956	0.0212	1.2744	0.21	86.6%	573.04%
	HAP	0.2122	1.2804	0.0347	1.2457	0.19	85.8%	503.39%

3.8 Swelling, absorption and dissolution assessment

Table S4: Dry scaffold mass W_d (g), weight of swollen scaffolds W_s (g) and swelling ratio SR_i (%) of the HAP-CeO_2 composite scaffolds and of HAP reference scaffold, after immersion into PBS solution (pH= 7.4, @37 °C) for i hours (i= 24, 48, 72).

Series	CeO_2 content (%)	W_d (g)	W_s (g) (@24 h)	SR_{24} (%) (@24 h)	W_s (g) (@48 h)	SR_{48} (%) (@48 h)	W_s (g) (@72 h)	SR_{72} (%) (@72 h)
1S	4 % CeO_2	0.3502	2.3697	576.67%	2.3313	565.71%	2.3233	563.42%
	4 % CeO_2	0.3101	2.1090	580.10%	2.0800	570.75%	2.0877	573.23%
	5 % CeO_2	0.3284	2.4044	632.16%	2.3702	621.74%	2.3663	620.55%
	5 % CeO_2	0.2963	2.1877	638.34%	2.1222	616.23%	2.0922	606.11%
	10 % CeO_2	0.1582	1.6528	944.75%	1.5733	894.50%	1.4989	847.47%
	10 % CeO_2	0.1474	1.4344	873.13%	1.3567	820.42%	1.3680	828.09%
2S	4 % CeO_2	0.2218	2.1584	873.13%	2.1145	853.34%	2.1123	852.34%
	4 % CeO_2	0.2102	2.1325	914.51%	2.0895	894.05%	2.0657	882.73%
	5 % CeO_2	0.1962	1.7869	810.75%	1.7567	795.36%	1.7424	788.07%
	5 % CeO_2	0.2115	1.9562	824.92%	1.9351	814.94%	1.9198	807.71%
	10 % CeO_2	0.1832	1.5327	736.63%	1.6866	820.63%	1.5903	768.07%
	10 % CeO_2	0.1681	1.4322	751.99%	1.4142	741.28%	1.4043	735.40%
3S	4 % CeO_2	0.1821	x*	-	x	-	x	-
	4 % CeO_2	0.1914	1.6763	775.81%	x	-	x	-
	5 % CeO_2	0.0845	0.5543	555.98%	0.5375	536.09%	0.5300	527.22%
	5 % CeO_2	0.2915	2.4321	734.34%	2.3981	722.68%	2.3767	715.33%
	10 % CeO_2	0.2654	2.1934	726.45%	2.1638	715.30%	x	-
	10 % CeO_2	0.2532	2.1454	747.31%	x	-	x	-
	HAP	0.2360	2.1513	811.57%	2.1113	794.62%	2.0096	751.53%
	HAP	0.2153	2.0154	836.09%	1.9843	821.64%	1.8567	762.38%

*Measurement was not recorded due to dissolution of the sample.

Table S5: Dry scaffold mass W_d (g), saturated weight W_{sat} (g) and absorption ratio AR_i (%) of the HAP-CeO_2 composite scaffolds and of HAP reference scaffold, after immersion into PBS solution (pH= 7.4, @37 °C) for i hours (i= 24, 48, 72).

series	CeO_2 content (%)	W_d (g)	W_{sat} (g) (@24 h)	AR_{24} (%) (@24 h)	W_{sat} (g) (@48 h)	AR_{48} (%) (@48 h)	W_{sat} (g) (@72 h)	AR_{72} (%) (@72 h)
1S	4 % CeO_2	0.3502	2.1139	503.63%	2.1071	501.68%	2.1018	500.17%
	4 % CeO_2	0.3101	1.8411	493.71%	1.8495	496.42%	1.8302	490.20%
	5 % CeO_2	0.3284	2.1775	563.06%	2.0820	533.98%	2.0973	538.64%
	5 % CeO_2	0.2963	1.8716	531.66%	1.7452	489.00%	1.8203	514.34%
	10 % CeO_2	0.1582	1.4028	786.73%	1.3253	737.74%	1.2612	697.22%
	10 % CeO_2	0.1474	1.1603	687.18%	1.0459	609.57%	1.0696	625.64%
2S	4 % CeO_2	0.2218	1.9124	762.22%	1.9024	757.71%	1.8974	755.46%
	4 % CeO_2	0.2102	1.9026	805.14%	1.8936	800.86%	1.8799	794.34%
	5 % CeO_2	0.1962	1.5669	698.62%	1.5549	692.51%	1.5349	682.31%
	5 % CeO_2	0.2115	1.7247	715.46%	1.7047	706.00%	1.6897	698.91%
	10 % CeO_2	0.1832	1.2275	570.03%	1.3355	628.98%	1.2316	572.27%
	10 % CeO_2	0.1681	1.2134	621.83%	1.2024	615.29%	1.1924	609.34%
3S	4 % CeO_2	0.1821	x*	-	x	-	x	-
	4 % CeO_2	0.1914	0.9495	396.08%	x	-	x	-
	5 % CeO_2	0.0845	0.4558	439.41%	0.4385	418.93%	0.4304	409.35%
	5 % CeO_2	0.2915	1.6567	468.34%	1.6378	461.85%	1.6244	457.26%
	10 % CeO_2	0.2654	1.5289	476.07%	1.5164	471.36%	x	-
	10 % CeO_2	0.2532	1.431	465.17%	x	-	x	-
	HAP	0.2360	1.7340	634.75%	1.7534	642.97%	1.7171	627.58%
	HAP	0.2153	1.5470	618.53%	1.5687	628.61%	1.5193	605.67%

*Measurement was not recorded due to dissolution of the sample.

Table S6: Average values of swelling ratio SRi (%) of the HAP-CeO_2 composite scaffolds and of HAP reference scaffold, after immersion into isopropanol for 5 minutes and average values of swelling ratio SRi (%) and absorption ratio ARi (%) after immersion into PBS solution (pH= 7.4, @37 °C) for 72 hours.

sample	SR_5 (%) (@5 min)	SR_{72} (%) (@72 h)	AR_{72} (%) (@72 h)
1S – 4 % CeO_2	431.18	568.32	495.19
1S – 5 % CeO_2	465.14	613.33	526.49
1S – 10 % CeO_2	695.38	837.78	661.43
2S – 4 % CeO_2	574.02	867.54	774.9
2S – 5 % CeO_2	637.87	797.89	690.61
2S – 10 % CeO_2	623.06	751.74	590.81
3S – 4 % CeO_2	393.16	-	-
3S – 5 % CeO_2	370.79	621.28	433.31
3S – 10 % CeO_2	382.99	-	-
HAP	538.22	756.96	616.63

Table S7: Dry scaffold mass W_d (g), dry scaffold mass after freeze-drying W_{fd} (g) and dissolution D (%) of the HAP-CeO_2 composite scaffolds and of HAP reference scaffold, after immersion into PBS solution (pH= 7.4, @37 °C) for 7 days.

series	CeO_2 content (%)	W_d (g)	Wf_d (g)	$W_d - W_{fd}$	D (%)
1S	4 % CeO_2	0.3502	0.2765	0.0737	21.05%
	4 % CeO_2	0.3101	0.2430	0.0671	21.64%
	5 % CeO_2	0.3284	0.2638	0.0646	19.67%
	5 % CeO_2	0.2963	0.2305	0.0658	22.21%
	10 % CeO_2	0.1582	0.1253	0.0329	20.80%
	10 % CeO_2	0.1474	x*	-	-
2S	4 % CeO_2	0.2218	0.1945	0.0273	12.31%
	4 % CeO_2	0.2102	0.1887	0.0215	10.23%
	5 % CeO_2	0.1962	0.1683	0.0279	14.22%
	5 % CeO_2	0.2115	0.1834	0.0281	13.29%
	10 % CeO_2	0.1832	0.1594	0.0238	12.99%
	10 % CeO_2	0.1681	0.1426	0.0255	15.17%
3S	4 % CeO_2	0.1821	x	-	-
	4 % CeO_2	0.1914	x	-	-
	5 % CeO_2	0.0845	0.0628	0.0217	25.68%
	5 % CeO_2	0.2915	0.2113	0.0802	27.51%
	10 % CeO_2	0.2654	x	-	-
	10 % CeO_2	0.2532	x	-	-
	HAP	0.2360	0.2251	0.0109	4.62%
	HAP	0.2153	0.2041	0.0112	5.20%

*Measurement was not recorded due to dissolution of the sample.

Figure S7：High-resolution TEM images

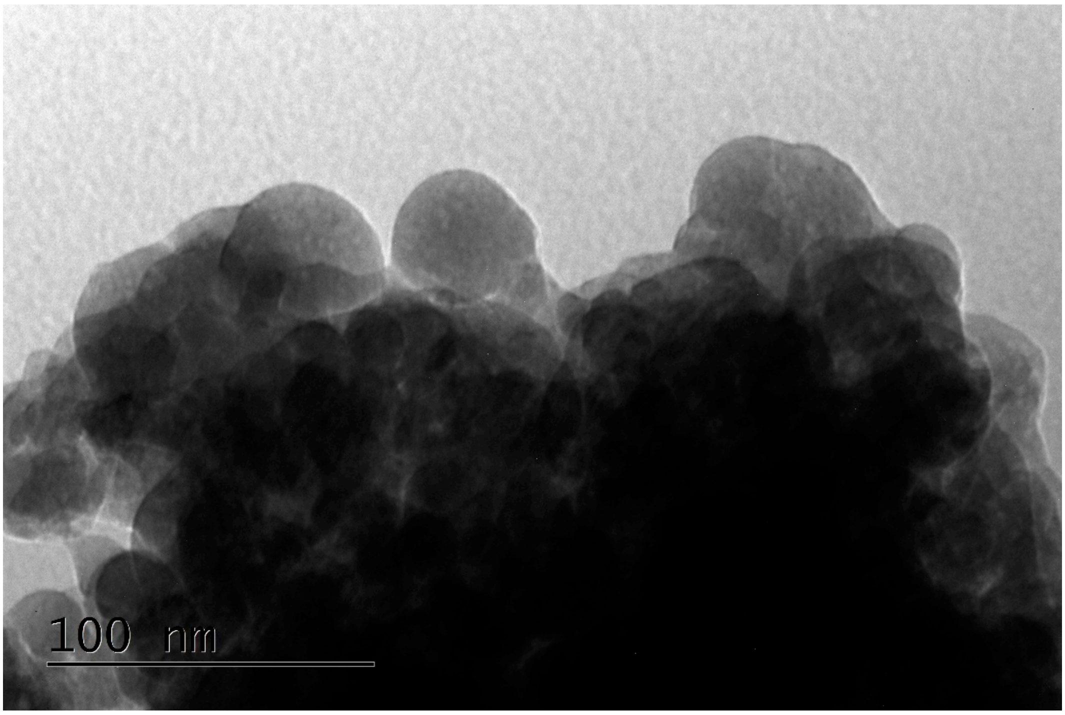

Figure 2a

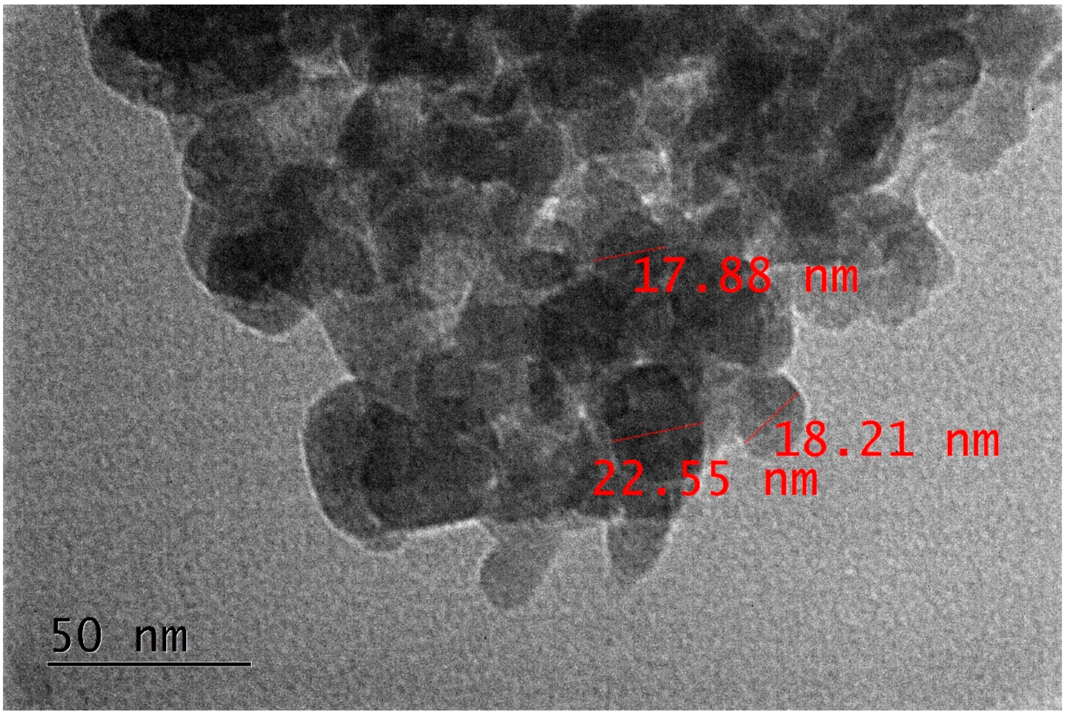

Figure 2b1

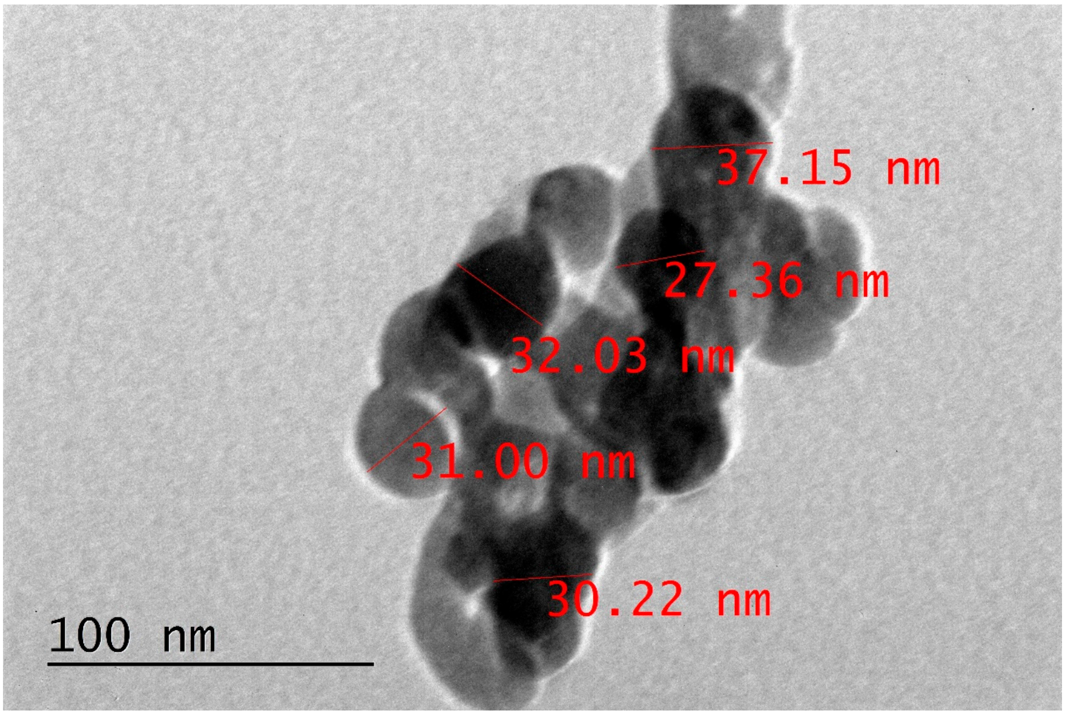

Figure 2b2

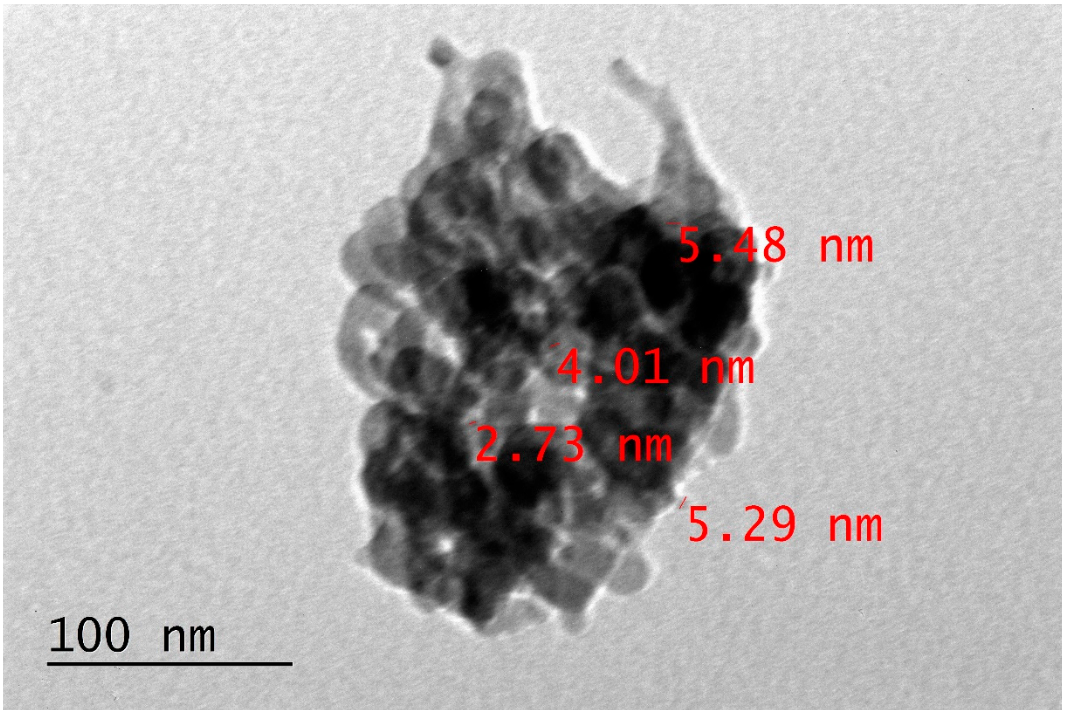

Figure 2c

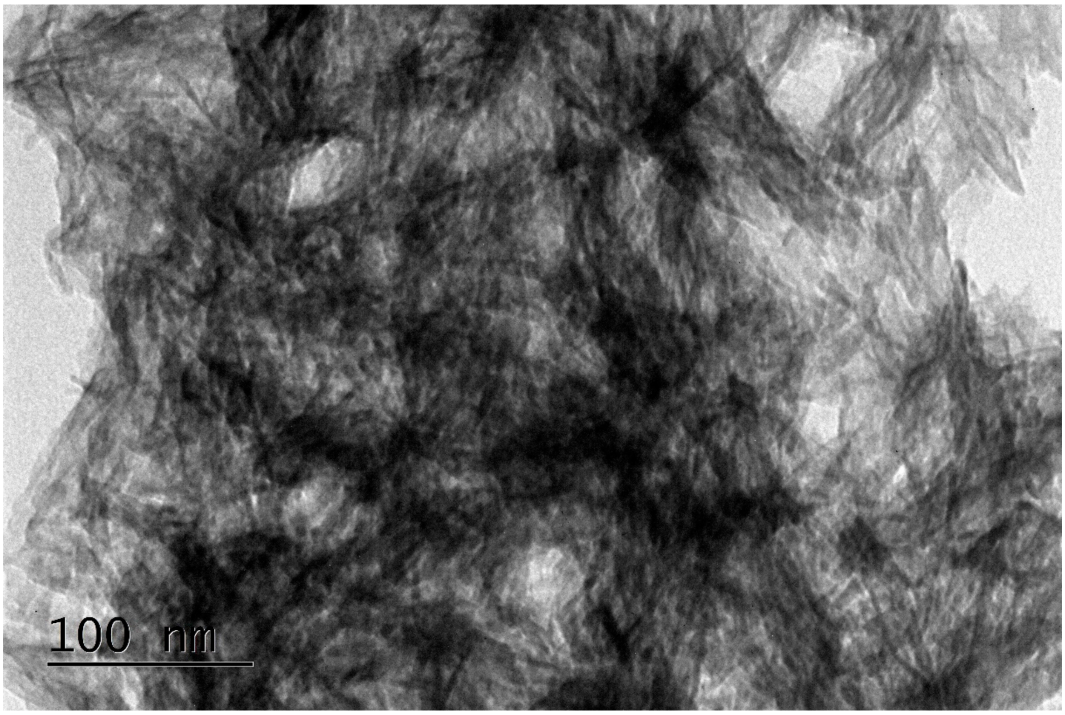

Figure 2d

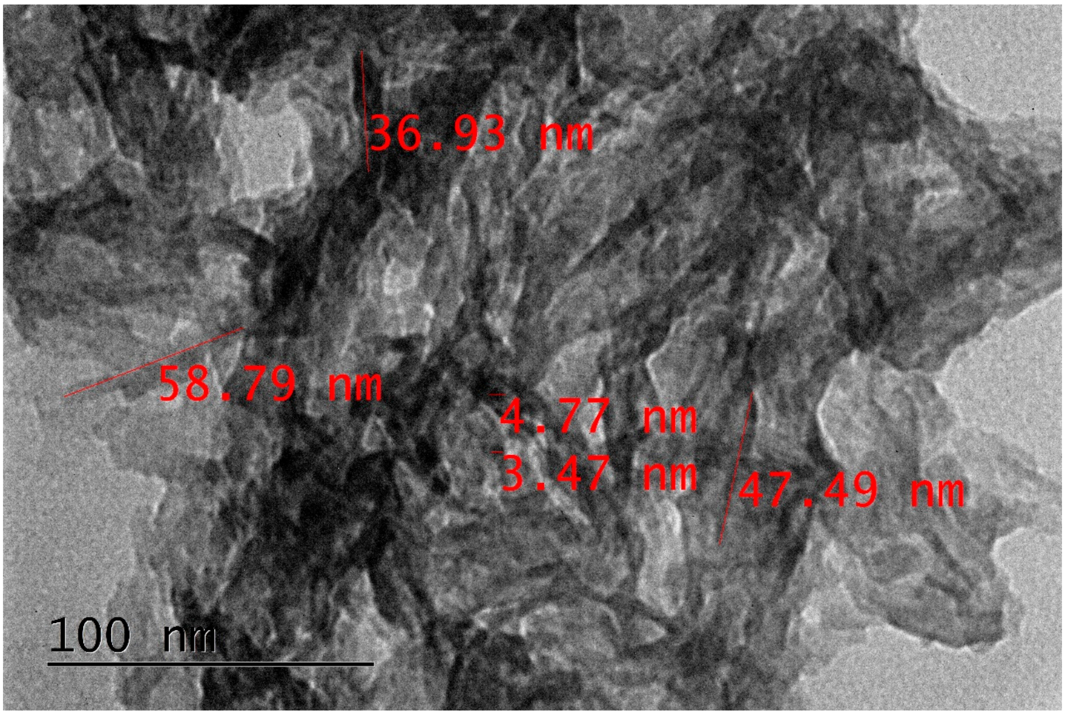

Figure 2e

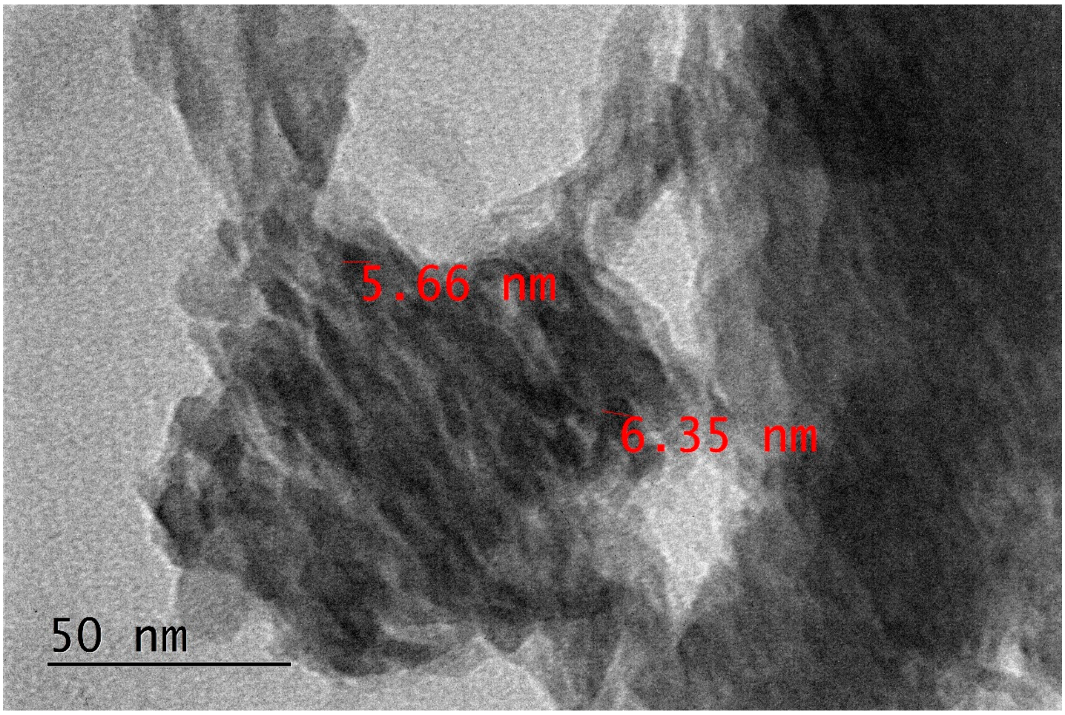

Figure 2f

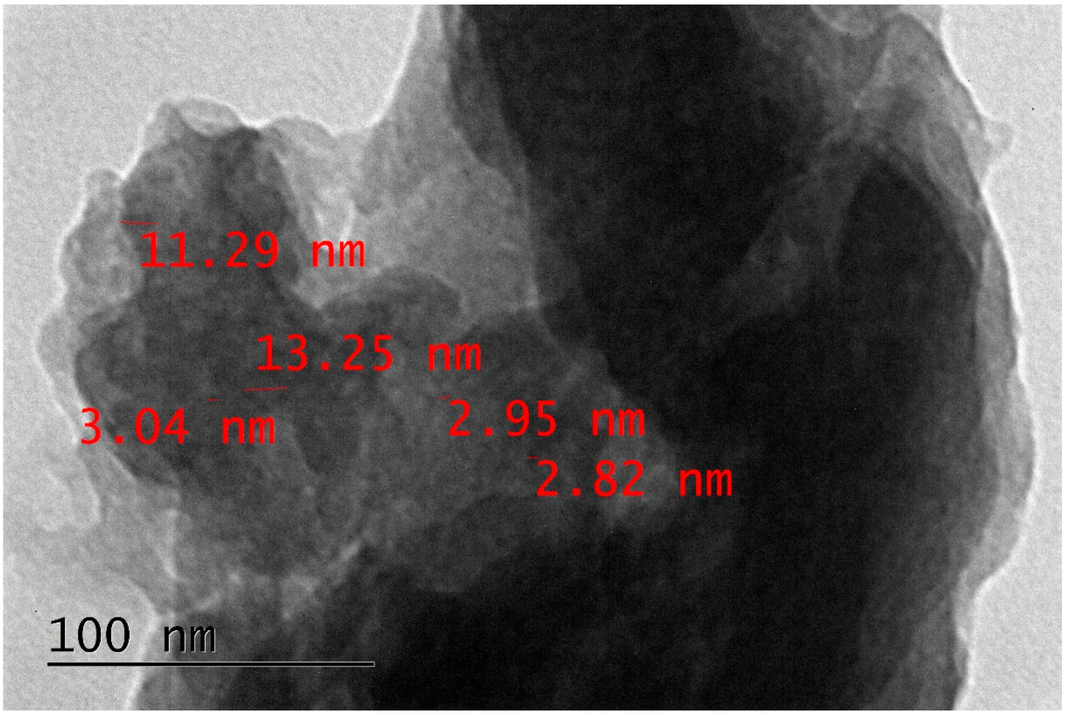

Figure 2g

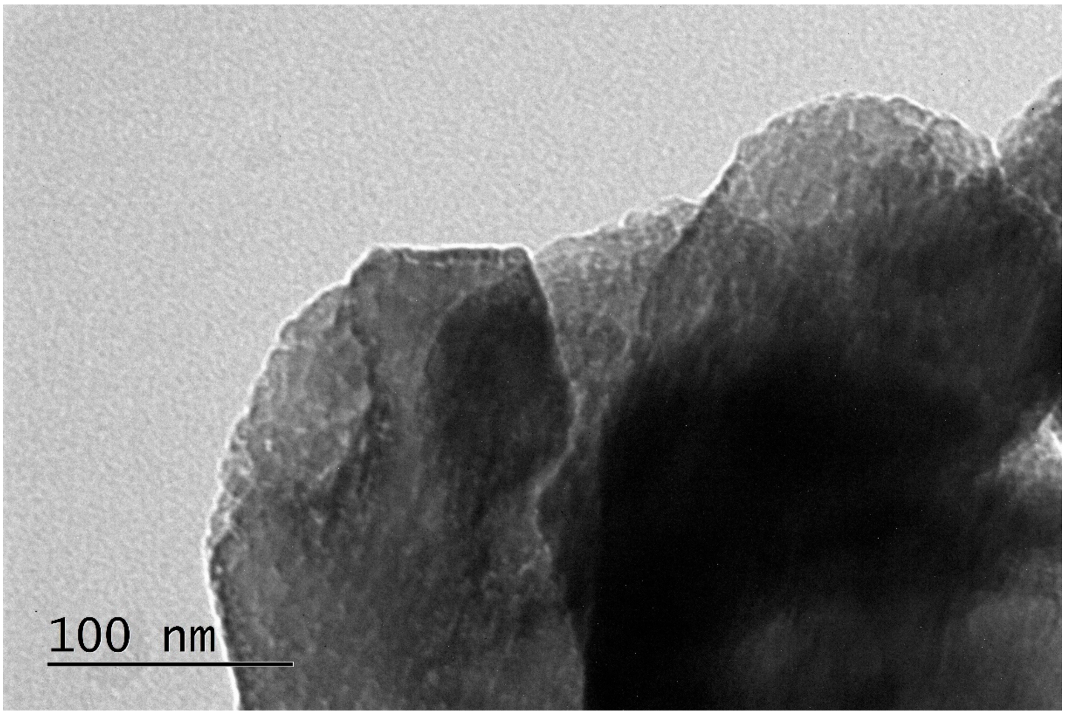

Figure 2h

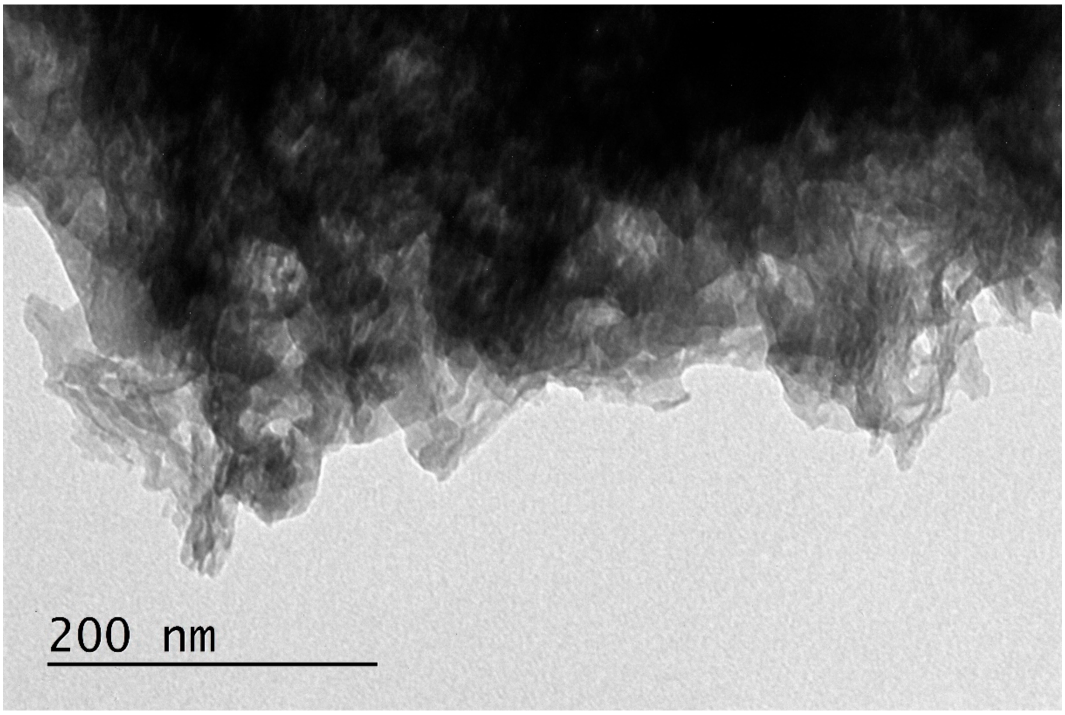

Figure 2i
